# The Phospholipid *N*-Methyltransferase and Phosphatidylcholine Synthase Pathways and the ChoXWV Choline Uptake System Involved in Phosphatidylcholine Synthesis Are Widely Conserved in Most, but Not All *Brucella* Species

**DOI:** 10.3389/fmicb.2021.614243

**Published:** 2021-08-04

**Authors:** Beatriz Aragón-Aranda, Leyre Palacios-Chaves, Miriam Salvador-Bescós, María Jesús de Miguel, Pilar M. Muñoz, Miguel Ángel Vences-Guzmán, Amaia Zúñiga-Ripa, Leticia Lázaro-Antón, Christian Sohlenkamp, Ignacio Moriyón, Maite Iriarte, Raquel Conde-Álvarez

**Affiliations:** ^1^Dpto. de Microbiología y Parasitología, Instituto de Salud Tropical (ISTUN), Instituto de Investigación Sanitaria de Navarra, Universidad de Navarra, Pamplona, Spain; ^2^Unidad de Producción y Sanidad Animal, Centro de Investigación y Tecnología Agroalimentaria de Aragón, Zaragoza, Spain; ^3^Instituto Agroalimentario de Aragón-IA2, CITA-Universidad de Zaragoza, Zaragoza, Spain; ^4^Centro de Ciencias Genómicas, Universidad Nacional Autónoma de México, Cuernavaca, Mexico

**Keywords:** brucella, phospholipid, phosphatidylcholine, choline, cell envelope

## Abstract

The brucellae are facultative intracellular bacteria with a cell envelope rich in phosphatidylcholine (PC). PC is abundant in eukaryotes but rare in prokaryotes, and it has been proposed that *Brucella* uses PC to mimic eukaryotic-like features and avoid innate immune responses in the host. Two PC synthesis pathways are known in prokaryotes: the PmtA-catalyzed trimethylation of phosphatidylethanolamine and the direct linkage of choline to CDP-diacylglycerol catalyzed by the PC synthase Pcs. Previous studies have reported that *B. abortus* and *B. melitensis* possess non-functional PmtAs and that PC is synthesized exclusively *via* Pcs in these strains. A putative choline transporter ChoXWV has also been linked to PC synthesis in *B. abortus*. Here, we report that Pcs and Pmt pathways are active in *B. suis* biovar 2 and that a bioinformatics analysis of *Brucella* genomes suggests that PmtA is only inactivated in *B. abortus* and *B. melitensis* strains. We also show that ChoXWV is active in *B. suis* biovar 2 and conserved in all brucellae except *B. canis* and *B. inopinata.* Unexpectedly, the experimentally verified ChoXWV dysfunction in *B. canis* did not abrogate PC synthesis in a PmtA-deficient mutant, which suggests the presence of an unknown mechanism for obtaining choline for the Pcs pathway in *Brucella*. We also found that ChoXWV dysfunction did not cause attenuation in *B. suis* biovar 2. The results of these studies are discussed with respect to the proposed role of PC in *Brucella* virulence and how differential use of the Pmt and Pcs pathways may influence the interactions of these bacteria with their mammalian hosts.

## Introduction

Brucellosis is a worldwide extended zoonosis caused by bacteria of the genus *Brucella*, a group of facultative intracellular pathogens belonging to the α-2 class of *Proteobacteria*. *B. melitensis, B. abortus*, and *B. suis* are the best-known *Brucella* species and they were divided long ago into biovars according to phenotypic criteria (Alton et al., 1988; Moreno, 2014). The *B. abortus* and *B. melitensis* biovars are genetically and phenotypically homogeneous and they predominantly infect cattle and small ruminants, respectively. The *B. suis* biovars, in contrast, exhibit greater phylogenomic diversity and broader host ranges (Moreno et al., 2002; Scholz et al., 2008; Whatmore, 2009; Al Dahouk et al., 2012). *B. suis* biovars 1 and 3, for instance, are endemic in America and Asia where they cause serious reproductive problems in pigs and severe disease in humans (EFSA, 2009). *B. suis* biovar 2 poses an important problem for pig farming in Europe (EFSA, 2009) where wild boars and hares act as reservoirs (Garin-Bastuji et al., 2000, 2006; Muñoz et al., 2010). *B. suis* biovar 4 infects reindeer in Eurasia and Alaska, and *B. suis* biovar 5 has been isolated from wild rodents in Transcaucasia (Zheludkov and Tsirelson, 2010). Other brucellae infect marine mammals (*B. pinnipedialis* and *B. ceti*), the American wood rat (*B. neotomae*), the European common vole (*B. microti*), domestic dogs (*B. canis*), and sheep (*B. ovis*) (Zheludkov and Tsirelson, 2010); in the case of *B. neotomae* and *B. canis*, human infections have also been reported (Suárez-Esquivel et al., 2017; Moreno, 2021). *B. inopinata*, *B. vulpis*, and *B. papionis* are species proposed for a few isolates respectively obtained from a breast implant (one strain), fox mandibular lymph nodes (2 strains), and abortion-related materials in captive baboons (two strains) (Scholz et al., 2010, 2016; Whatmore et al., 2014). All these species except *B. inopinata* form a core group separated from early diverging clades closer to other α-2 *Proteobacteria*. Early diverging brucellae include *B. inopinata* and unnamed *Brucella* isolates from Australian rodents and frogs (Wattam et al., 2014; Soler-Lloréns et al., 2016; Al Dahouk et al., 2017).

Investigations carried out mostly with strains representative of biovars 1 of *B. melitensis, B. abortus*, and *B. suis* show that their pathogenicity is largely linked to deficient detection by the host innate immune system during the early stages of infection. The subsequent delay in induction of the inflammatory response allows these bacteria to reach safe intracellular niches where they multiply and establish long-lasting infections (Barquero-Calvo et al., 2007, 2009; Martirosyan et al., 2011). *Brucella* cell envelope (CE) components such as the lipopolysaccharide (LPS) and lipoproteins as well as the overall CE architecture do not bear the typical pathogen-associated molecular patterns (PAMPs) that in other bacteria are recognized by Toll-like receptors (TLRs) and targeted by bactericidal peptides and the antibody-independent complement cascade. Instead, the CE of brucellae has an atypical LPS, peculiar amino lipids, and large amounts of phosphatidylcholine (PC) (Gamazo and Moriyon, 1987; Parent et al., 2007; Martirosyan et al., 2011; Palacios-Chaves et al., 2011). This typically eukaryotic phospholipid, while scarcely present in most bacterial groups (Aktas et al., 2010), is common in the α-2 *Proteobacteria.* This phylogenetic group includes *Brucella* and the plant pathogens and symbionts *Agrobacterium* and *Sinorhizobium*, which live in close association with eukaryotic cells (Moreno, 1992). The observation that *Brucella* and *Agrobacterium* mutants lacking PC in their cell envelopes are defective in their interactions with their respective eukaryotic hosts has led to the proposition that this phospholipid plays an important role in allowing these bacteria to avoid recognition by host defenses (Comerci et al., 2006; Conde-Álvarez et al., 2006; Aktas et al., 2010).

**FIGURE 1:**
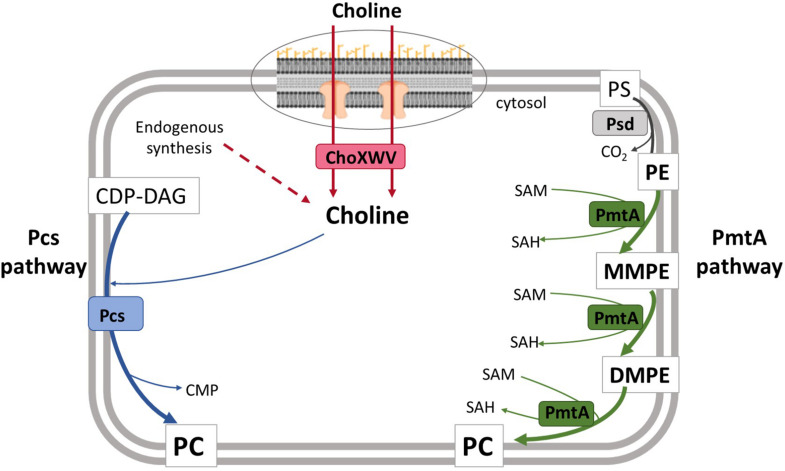
Model for phosphatidylcholine biosynthesis in *B. suis* biovar 2. In the Pcs pathway, the membrane protein Pcs condenses CDP-DAG and choline to generate PC. This choline is taken up by the ChoXWV transporter. Dashed arrows indicate steps for which putative genes have not been identified in *Brucella*. In the PmtA pathway, three successive methylations catalyzed by PmtA convert PE to PC. PmtA, phospholipid N-methyltransferase; Pcs, phosphatidylcholine synthase; ChoXWV, high-affinity choline transport system; SAM, *S-*adenosylmethionine; SAH, *S*-adenosylhomocysteine, CMP; cytidine monophosphate; CDP-DAG, cytidine diphosphate diacylglycerol; PS, phosphatidylserine; PE, phosphatidylethanolamine; MMPE, monomethylphosphatidylethanolamine; DMPE, dimethylphosphatidylethanolamine; PC, phosphatidylcholine.

Whereas in eukaryotes PC synthesis occurs through the CDP-choline pathway (also called the Kennedy pathway), PC can be synthesized in bacteria by either the phospholipid N-methylation (Pmt) pathway or the phosphatidylcholine synthase (Pcs) pathway (Sohlenkamp et al., 2000, 2003; Aktas et al., 2010) (Figure 1). The Pmt pathway consists of the stepwise methylation of phosphatidylethanolamine (PE) sequentially generating monomethyl-PE (MMPE), dimethyl-PE (DMPE), and PC (Sohlenkamp et al., 2003; Aktas et al., 2010; Geiger et al., 2013). These three steps are catalyzed by a single cytosolic phospholipid *N*-methyltransferase (PmtA) that uses *S*-adenosylmethionine (SAM) as the methyl donor (Sohlenkamp et al., 2003), although other methyltransferases (PmtX) capable of catalyzing this reaction have been described in *Bradyrhizobium* (Aktas et al., 2010). The choline-dependent Pcs pathway consists of the condensation of this quaternary amine with CDP-diacylglycerol (CDP-DAG) catalyzed by Pcs (Sohlenkamp et al., 2000). Both pathways are active in *Agrobacterium* and *Sinorhizobium* (Aktas et al., 2010), but previous studies have reported that only the Pcs pathway is functional in *B. abortus* and *B. melitensis* due to the presence of mutated *pmtA* alleles in these strains (Martínez-Morales et al., 2003; Comerci et al., 2006; Aktas et al., 2010). Accordingly, the choline transporter ChoXWV1 is also required for PC biosynthesis in *B. abortus* (Herrmann et al., 2013).

In view of the importance of PC biosynthesis for the wild-type virulence of *B. abortus* (Comerci et al., 2006; Conde-Álvarez et al., 2006), we thought it worthwhile to investigate the PC biosynthesis pathways and ChoXWV choline uptake systems in other *Brucella* spp. Our experimental findings indicate that most *Brucella* strains excluding *B. abortus* and *B. melitensis* have the genetic capacity to carry out PC synthesis using either the Pmt or Pcs pathway, and all but *B. canis* and *B. inopinata* strains encode functional choline transporters. Remarkably, our studies provide evidence that *B. canis* can utilize the choline-dependent Pcs pathway in the absence of a functional high-affinity choline transporter, which suggests the presence of an uncharacterized choline biosynthesis pathway in these bacteria.

## Materials and Methods

### Bacterial Strains and Growth Conditions

The characteristics of the bacterial strains and plasmids are shown in Supplementary Table 1. The virulent *B. suis* biovar 2 (bv2) strain CITA 198 (henceforth Bs2WT) was selected for this study because the reference bv2 strain *B. suis* Thomsen is attenuated in mice (Aragón-Aranda et al., 2020). The choline-containing media used were tryptic soy broth (TSB, Scharlau, Barcelona, Spain), the same medium with 15% agar (TSA) or Blood Agar Base (BAB; Oxoid, United Kingdom), supplemented when necessary with 50 μg/ml kanamycin (Km), or/and 5% sucrose or/and 0.2% activated charcoal (Sigma) (see below). The choline-free medium was the lactate-glutamate-glycerol-vitamins medium of Gerhardt (1958) supplemented with 1 mM methionine (a Bs2WT requirement; Zúñiga-Ripa, unpublished results) (henceforth mGSM). *E. coli* strains were cultured on TSA-Km (50 μg/ml), supplemented with 1 mM diaminopimelic acid (DAP [Sigma]) in the case of *E. coli* β2150. All strains were stored at −80°C either in skimmed milk (Scharlau, Barcelona, Spain) or in TSB containing 0.5% yeast extract and 7% dimethylsulfoxide. Work was performed under Biosafety Level 3 (BSL-3) conditions.

### Sequence Analyses

Genomic sequences of *B. suis* bv2 Thomsen (ATCC 23445 or NCBI:txid470137), *B. melitensis* bv1 16M (ATCC 23456 or NCBI:txid224914), *B. abortus* bv1 2308 (NCBI:txid359391), and *S. meliloti* 1021 (NCBI:txid266834) were obtained from the databases at National Center for Biotechnology Information (NCBI) or Kyoto Encyclopedia of Genes and Genomes (KEGG). For Bs2WT (genomic sequence not available), the ORF of interest were PCR amplified and then sequenced at “Servicio de Secuenciación del Centro de Investigación Médica Aplicada” (CIMA, Pamplona, Spain). Sequence alignments were performed with Clustal Omega.

### DNA Manipulations and Construction of Mutants

Plasmids and chromosomal DNA were extracted with QIAprep^®^ Spin Miniprep Kit and QIAamp^®^ DNA Miniprep Kit (Qiagen GmbH, Hilden, Germany). When needed, DNA was purified from agarose gels using a QIAquick Gel extraction Kit (Qiagen GmbH, Hilden, Germany). Primers were synthesized by Sigma-Genosys Ltd. (Haverhill, United Kingdom).

Bs2WT in-frame deletion mutants encompassing the consensus sequences of *pcs* and *pmtA* were constructed using plasmids pRCI-22 and pRCI-10, respectively (Conde-Álvarez et al., 2006). These suicide plasmids were extracted from *E. coli* TOP10F′ and transformed into the DAP auxotrophic *E. coli* β2150 donor strain (Allard et al., 2015). To obtain the Bs2Δ*pcs* mutant, pRCI-22 was introduced into Bs2WT by tri-parental mating with *E. coli* β2150 (carrying the suicide plasmid) and *E. coli* β2150-pRK2013 (helper strain) (Figurski and Helinski, 1979). The first recombinant was selected by Km resistance and DAP independence on media with charcoal. Bacteria resulting from allelic exchange by double recombination were selected on sucrose, and the loss of the plasmid was confirmed by Km sensitivity. The same strategy was followed to construct Bs2Δ*pmtA* using suicide plasmid pRCI-10 and the double mutant Bs2Δ*pcs*Δ*pmtA* by deletion of *pmtA* in Bs2Δ*pcs*. The loss of the plasmid concomitant with the gene deletion in each of these mutants was confirmed by PCR (oligonucleotides in Supplementary Table 2). The Bs2Δ*pcs*Δ*pmtA_pcs* and Bs2Δ*pcs*Δ*pmtA_pmtA* complemented strains were constructed by introduction of pRCI-40 (Conde-Álvarez et al., 2006) and pBA-8 in Bs2Δ*pcs*Δ*pmtA*, respectively. To generate the pBA-8 plasmid, pRCI-41 (Conde-Álvarez et al., 2006) was modified at position G62D by PCR site-directed mutagenesis (Supplementary Table 2) to restore the PmtA consensus motif [VL(E/D)XGXGXG] present in *B. suis* bv2.

The suicide plasmid used to generate in-frame deletions in *choX1* was constructed by PCR overlap using *B. abortus* 2308 genomic DNA as the template. Oligonucleotides *choX1*-F1 and *choX1*-R2 (Supplementary Table 2) were used to amplify a 493-base pair (bp) fragment including codons 1 to 54 of the BAB1_1593 homolog and 330 bp upstream of its first putative start codon, and primers *choX1*-F3 and *choX1*-R4 (Supplementary Table 2) were used to amplify a second fragment of 770 bp including codons 253 to 322 of BAB1_1593 and 555 bp downstream of its stop codon. These two fragments were ligated by overlapping PCR using primers *choX1-*F1 and *choX1*-R4 for amplification and the complementary regions between *choX2*-R2 and *choX1*-F3 for overlapping. The new fragment (containing the deletion allele) was cloned into pCR2.1 (Invitrogen) to generate pLPI-1. Plasmid pLPI was sequenced to confirm that the reading frame was maintained and then subcloned into the *Xba*I and the *Bam*HI sites of plasmid pJQK (Scupham and Triplett, 1997) to produce pLPI-2. This suicide plasmid was introduced into Bs2WT by conjugation as described above. The mutant generated by allelic exchange was selected by Km sensitivity and sucrose resistance and confirmed by PCR using the corresponding oligonucleotides (Supplementary Table 2).

Bs2Δ*choX* was constructed using the same methodology. Oligonucleotides *choX2*-F1 and *choX2*-R2 (Supplementary Table 2) were used to amplify a 446-bp fragment including codons 1 to 3 of the BAB2_0502 homolog and 437 bp upstream of its start codon, and oligonucleotides *choX2*-F3 and *choX2*-R4 (Supplementary Table 2) were used to amplify a second 494-bp fragment including codons 268 to 288 of BAB2_0502 and 428 bp downstream of the stop codon. These two fragments were ligated using primers *choX2-*F1 and *choX2*-R4, and the new fragment containing the deletion allele was cloned into pCR2.1 to generate pLPI-8. After sequencing to confirm that the reading frame was maintained, pLPI-8 was subcloned in pJQK to produce the mutator plasmid pLPI-9. Plasmid pLPI-9 was used to construct Bs2Δ*choX2* and Bs2Δ*choX1*Δ*choX2* using Bs2WT and Bs2Δ*choX1* as background, respectively. After conjugation with Bs2WT, the resulting colonies were screened by PCR (Supplementary Table 2) to identify the *choX2*-deleted mutants. Similarly, mutant Bs2Δ*pmtA*Δ*choX1*Δ*choX2* was constructed on Bs2Δ*pmtA* by sequential use of plasmids pLPI-2 and pLPI-9. The Bs2Δ*choX1_choX1* complemented strain was constructed by stable insertion of the miniTn7 transposon into the chromosome of Bs2Δ*choX1* (Choi et al., 2005). To generate the pBA-13 plasmid, we first generated a PCR product using oligonucleotides *choX*1_Fw_Tn7 and *choX*1_Rv_Tn7 (Supplementary Table 2). This PCR product was cloned into the corresponding sites of the linearized pUC18 R6KT miniTn7T Km^*R*^ vector (Llobet et al., 2009). The plasmid was introduced into *E. coli* S17 and transferred to Bs2Δ*choX*1 mutant by tetra-parental conjugation between *E. coli* S17 (pBA-13), *E. coli* SM10 λpir (pTNS2), and *E. coli* HB101 (pRK2013). The construct (Bs2Δ*choX1_choX1*) was examined by PCR for the correct insertion and orientation of the transposon between the *glmS* and *recG* genes as previously described (Martínez-Gómez et al., 2018). Finally, BcΔ*pmtA* was constructed by in-frame deletion of *pmtA* using pRCI-10 as described for Bs2WT.

### Growth Curves

Growth curves were obtained in a Bioscreen C (Lab Systems) apparatus. When mGSM was used, the inocula were prepared in this choline-free medium as follows. First, bacteria were grown in 10 ml of TSB at 37°C with orbital shaking for 18 h, harvested by centrifugation, resuspended in 10 ml of mGSM up to an optical density at 600 nm (O.D._600 *nm*_) of 0.1, and incubated at 37°C with orbital shaking. These exponentially growing bacteria were harvested by centrifugation, resuspended at an O.D._600 *nm*_ of 0.1 in the same medium and 200 μl/well aliquots dispensed as technical replicates in Bioscreen C multi-well plates. Plates were incubated with continuous shaking at 37°C for 5 days and absorbance values at 420–580 nm recorded every 30 min. Growth curves in TSB were obtained following a similar protocol but the exponential phase inoculum was prepared in TSB. All experiments were repeated at least three times. Controls with medium and no bacteria were included in all experiments.

### Choline Transport Assay

Transport assays were performed as described by Dupont et al. (2004) with minor modifications. Cells were grown in 10 ml of TSB at 37°C with orbital shaking until they reached the exponential phase (O.D._600 *nm*_ = 0.7), harvested by centrifugation, resuspended in mGSM at an O.D._600 *nm*_ of 1.0, and 1 ml of this suspension was mixed with 9 ml of this choline-free medium. When bacteria reached the exponential phase (O.D._600 *nm*_ = 0.5), they were washed twice with fresh mGSM and adjusted to an O.D._600 *nm*_ of 0.8. Transport assays were carried out using 1 ml of this cell suspension, with [methyl-^3^H]-choline chloride (82 Ci/mmol; Perkin Elmer) at a final concentration of 0.04 μM (optimized in preliminary experiments). After 15 and 60 min with continuous shaking at 37°C, bacteria were inactivated with phenol (0.5% final concentration) for 30 min at 37°C, collected on 22-μm membrane MF-Millipore (Sigma) filters using a 25-mm Swinnex filter holder (Merck). The filters were washed twice with 1 ml of fresh mGSM and the radioactivity in bacteria retained on the filter was measured in 2 ml of AquaLight (Hidex; Tecnasa) scintillation cocktail by a Hidex 300 SL Automatic TDCR Liquid Scintillation Counter (Gammadata Instrument AB, Uppsala, Sweden). Accumulation was expressed as μM of choline taking advantage of the efficiency correction (i.e., measurements in disintegrations per minute [d.p.m.]) of the apparatus. As a negative control, 1% phenol inactivated Bs2WT cells were used. All experiments were repeated at least twice.

### Lipid Analyses

The free-lipid fraction was extracted as described by Bligh and Dyer (1959) and analyzed by high-performance thin-layer chromatography (HPTLC) on silica gel 60 plates (Merck). The plates were pre-run with *n*-propyl alcohol:propionic acid:chloroform:water (3:2:2:1, vol/vol) and dried thoroughly before sample loading. Chromatography was performed in the same mixture of solvents (Higgins and Fieldsend, 1987) and plates were developed by charring at 180°C with 15% sulfuric acid in ethanol or with 10% CuSO_4_ in 8% phosphoric acid. L-β,γ-Dipalmitoyl-α cephalin (phosphatidylethanolamine) (Fluka A.G. Buch, Switzerland) and L-α-phosphatidylcholine distearoyl (Sigma Chemical Co., St. Louis, MO, United States) were used as standards.

The incorporation of choline into PC was studied in both TSB and mGSM. For TSB, a loopful of bacteria grown on TSA was inoculated in 20 ml of TSB, grown overnight, and an aliquot of the culture was resuspended in 20 ml of either TSB or mGSM to an O.D._600 *nm*_ of 0.2. This broth was supplemented with 6.6 μCi of [methyl-^3^H]-choline chloride (82 Ci/mmol; Perkin Elmer) and incubated at 37°C with stirring until the middle exponential phase (O.D._600 *nm*_ of 0.8). Then, cells were harvested by centrifugation and free lipids were analyzed as described above. Free lipids (extracted as described above) were chromatographed and the HPTLC plates were exposed to Biomax Kodak films at −80°C for 20 days. Moreover, the radioactivity in the appropriate phospholipid spots was quantified as follows. Plates were developed by charring with 10% CuSO_4_ in 8% phosphoric acid, the phospholipid spots were scraped, and the silica was collected. After washing the powder with methanol, the phospholipid was extracted with 1:2 chloroform:methanol and 2:1 chloroform:methanol (Guijas et al., 2016), the solvent was evaporated, and the extract was suspended in 2 ml of AquaLight (Hidex; Tecnasa) scintillation cocktail. Radioactivity was quantified as described above.

Radioactive labeling of free lipids with acetate was performed similarly using 6.6 μCi of sodium [1,2^–14^C] acetate (52 mCi/mmol; PerkinElmer) in 20 ml. Cells were grown, lipids were extracted, and autoradiography was performed as described above.

### Mouse Experiments

Seven-week-old female BALB/c mice (ENVIGO, Harlan) were lodged in cages in a BSL-3 facility (ES/31-2010-000132) with water and food *ad libitum* for 2 weeks before and during the experiments. For virulence assessment, groups of 10 mice were inoculated with the mutants or Bs2WT as parental strain control. Inocula were prepared by harvesting BAB grown bacteria in sterile buffered saline (BSS; 0.85% NaCl, 0.1% KH_2_PO_4_, and 0.2% K_2_HPO_4_; pH 6.85) and adjusting the suspension to 1 × 10^6^ CFU/ml. Mice were inoculated intraperitoneally with 1 × 10^5^ CFU in 0.1 ml (the exact dose was determined retrospectively on triplicate BAB plates). Two and 8 weeks after inoculation, mice were euthanized; the spleens were aseptically removed, individually weighed, and homogenized in 9 volumes of BSS; and serial 10-fold dilutions were plated by triplicate on BAB plates. After 5 days at 37°C, CFU were counted and the identity was confirmed by PCR. The data (mean CFU/spleen) were normalized by logarithmic transformation and the mean log_10_ CFU/spleen values and standard deviations (SDs) were calculated. These values were used for plotting and for statistical comparisons by one-way ANOVA and Fisher’s Protected Least Significant Differences (PLSD) tests (statistical procedures established for virulence comparisons in the mouse model of brucellosis, Grilló et al., 2012).

The procedures were in accordance with the current European (directive 86/609/EEC) and Spanish (RD 53/2013) legislations, supervised by both Ethical Committee for Animal Experimentation of CITA and Animal Welfare Committee of the University of Navarra and authorized by Aragón (reports No. 2014-20 and 2014-21) and Navarra (CEEA 045/12) Governments.

## Results

### Phosphatidylcholine Synthesis in *B. suis* Biovar 2 Takes Place Through Both the Methylation (PmtA) Pathway and the PC Synthase (Pcs) Pathway

**FIGURE 2:**
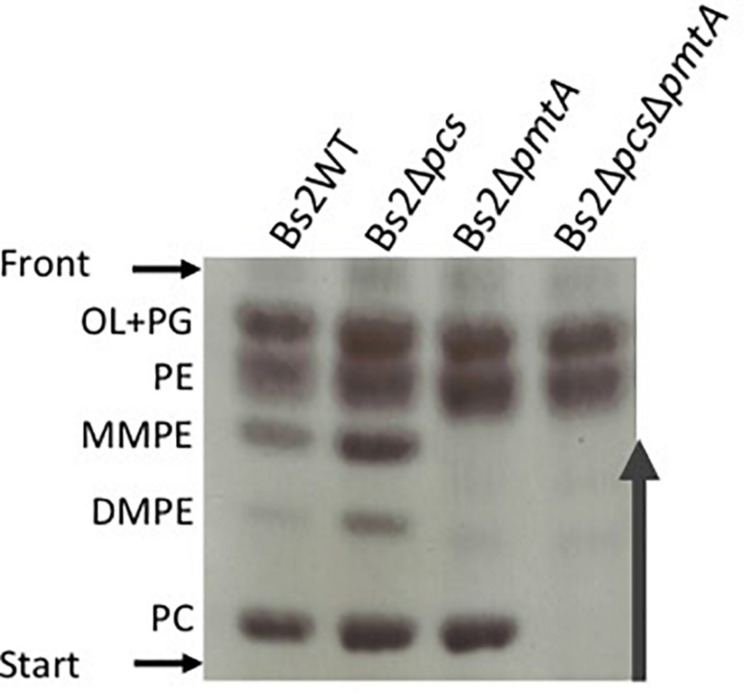
Phosphatidylcholine synthesis in Bs2WT takes place through the methylation (PmtA) and the phosphatidylcholine synthase pathway (Pcs). HPTLC analysis of the free lipids of Bs2WT, Bs2Δ*pcs*, Bs2Δ*pmtA*, and Bs2Δ*pcs*Δ*pmtA* grown in TSB. OL, ornithine lipids; PG, phosphatidylglycerol; PE, phosphatidylethanolamine; MMPE, mono-methyl-phosphatidylethanolamine; DMPE, dimethyl-phosphatidylethanolamine; PC, phosphatidylcholine.

We first assessed whether the synthesis of PC in Bs2WT was dependent on the PmtA and/or the Pcs pathways (Figure 1). To this end, we constructed single (Bs2Δ*pmtA* and Bs2Δ*pcs*) and double (Bs2Δ*pcs*Δ*pmtA*) non-polar mutants by making extensive in-frame deletions encompassing the consensus motifs associated with PmtA function. We then obtained stationary phase bacteria in TSB, extracted the free-lipid fractions and compared their composition by HPTLC. As shown in Figure 2, whereas Bs2WT and the single mutants Bs2Δ*pcs* and Bs2Δ*pmtA* produced similar amounts of PC, Bs2Δ*pcs*Δ*pmtA* did not produce PC in TSB. Moreover, we observed MMPE and DMPE (the PC precursors that demonstrate the activity of PmtA) only for Bs2WT and Bs2Δ*pcs*. When we complemented Bs2Δ*pcs*Δ*pmtA* with a plasmid carrying *pcs*, PC production was restored (Supplementary Figure 1). Introduction of a plasmid carrying *pmtA* (with an intact consensus sequence) into Bs2Δ*pcs*Δ*pmtA* resulted in MMPE and DMPE and PC production, although at lower levels than those in Bs2WT (Supplementary Figure 1). These observations show that PC synthesis in Bs2WT can take place through both the PmtA and Pcs pathways.

An intact Pcs pathway is required for the wild-type growth of *B. abortus* 2308 in a rich medium (Comerci et al., 2006; Conde-Álvarez et al., 2006; Supplementary Figure 2). In contrast, both Bs2Δ*pmtA* and Bs2Δ*pcs* grew normally in TSB, but the Bs2Δ*pcs*Δ*pmtA* mutant displayed slightly retarded growth in this medium (Supplementary Figure 2). These results are consistent with the functionality of both the PmtA and Pcs pathways in Bs2WT.

### Phospholipid *N*-Methyltransferase Is Conserved in *Brucella* Species Other Than *B. abortus* and *B. melitensis*

To better understand the differences in the PC synthesis pathways of Bs2WT and those reported for *B. abortus* 2308 and *B. melitensis*, and to explore whether these differences extend to other *Brucella* species, we carried out genomic comparisons. In the *B. suis* bv2 reference strain Thomsen genome sequence, BSUIS_A1967 is annotated as encoding the phospholipid *N*-methyltransferase PmtA, and we confirmed this annotation by sequencing the homologous ORF of Bs2WT. In the subsequent analyses, we found that the mutations identified previously in the SAM binding sites of the PmtAs encoded by *B. abortus* 2308 and *B. melitensis* 16M (Comerci et al., 2006; Conde-Álvarez et al., 2006) were not present in *B. suis* bv2 Thomsen or Bs2WT (Supplementary Figure 3). Moreover, when we examined the genome sequences of other *Brucella* strains representing different species and biovars, we found that all of the *B. melitensis* and *B. abortus* strains examined encode *pmtA* alleles carrying the same mutations described in *B. abortus* 2308 and *B. melitensis* 16M. In contrast, the genomes of type strains of *B. canis*, *B. microti, B. vulpis, B. ovis, B. ceti*, and *B. pinnipedialis*, and all of the genomes of the *B. suis* strains examined except *B. suis* bv1 1330 encode *pmtA* genes with conserved SAM binding motifs [VL(E/D)XGXGXG] (de Rudder et al., 2000; Sohlenkamp et al., 2003) (Supplementary Table 3). This polymorphism in the SAM binding motif of *B. suis* bv1 1330 was reported previously (Comerci et al., 2006) and, interestingly, is found in only one of the two available *B. suis* bv1 1330 genome sequences. No differences exist in the consensus motif (Sohlenkamp et al., 2000) of Pcs of all the *Brucella* spp. and biovars studied (Supplementary Table 4).

### Choline Transport Is Active in *B. suis* Biovar 2

Since the ChoXWV system is important for choline-dependent PC synthesis in *B. abortus* 2308 (Herrmann et al., 2013), we studied its functionality and connection with PC synthesis in Bs2WT. To this end, we first searched for orthologs of the *B. abortus* 2308 ChoXWV genes in *B. suis* bv2 Thomsen. We identified three operons: ORFs BSUIS_A1635 to 1637 (henceforth ChoXWV1) on chromosome I, BSUIS_B0730 to 0732 (henceforth ChoXWV2) on chromosome II, and BSUIS_A0222 to 0225 on chromosome I. Each of these operons contained orthologs of *choX*, *choW*, and *choV*, respectively, annotated as encoding a choline-glycine betaine-proline binding protein, a permease, and an ATP-binding protein (Aktas et al., 2011; Supplementary Figure 4). *S. meliloti* ChoX has high affinity for choline (equilibrium dissociation constant *K*_*D*_ 2.3–2.7 μM), only medium affinity for acetylcholine (*K*_*D*_ 100–145 μM) (Dupont et al., 2004; Oswald et al., 2008), and binds no betaine (Dupont et al., 2004). Analogous studies in *A. tumefaciens* found *K*_*D*_ values for choline, acetylcholine, and betaine of 2, 80, and 470 μM, respectively (Aktas et al., 2011). Similarly, the ChoX1 homolog in *B. abortus* shows dissociation constants of 7 ± 2 nM for choline and 350 ± 30 μM for betaine, and no specific L-carnitine or L-proline binding (Herrmann et al., 2013). While these types of experiments have not been conducted with *B. abortus* ChoX2, ligand binding assays performed with *B. abortus* YehZ (the protein encoded by the homolog of BSUIS_A0222) showed no interaction with glycine betaine, choline, ectoine, or carnitine at low ligand concentration, and only low affinity for glycine betaine (Herrou et al., 2017).

The crystal structure of *S. meliloti* ChoX shows two domains connected by two β-strands that conform a single functional linker in which a glycine residue (Gly^116^) flanked by alanine is thought to play an important role in substrate binding (Oswald et al., 2008). The amino acid sequence of the *B. suis* bv2 Thomsen ChoX1 and ChoX2 homologs showed that, although both have a Gly^120^, only ChoX1 has a flanking Ala^121^. Also, whereas both *B. suis* bv2 Thomsen ChoX1 and ChoX2 contain the Asp^45^ residue that forms a salt bridge with the choline head group in *S. meliloti*, only ChoX1 contains the Asn^156^–Asp^157^ residues that form hydrogen bonds with the tail of choline in *S. meliloti* ChoX (Supplementary Figure 5). Other amino acids involved in creating the ChoX–choline complex in *S. meliloti* are Cys^33^–Cys^247^, which form a disulfide bridge, and Met^91^–Pro^92^ and Glu^206^–Pro^207^, which form two *cis-*peptide bonds. In *B. suis* bv2 Thomsen, a disulfide bridge in a similar position (Cys^37^–Cys^251^) was presented only in ChoX1 (Supplementary Figure 5). Concerning the two *cis*-peptide bonds, the *Brucella* ChoX1 has Asn^95^–Pro^96^ and Glu^210^–Pro^211^, while ChoX2 presents Leu^87^–Pro^88^ and Ser^200^–Pro^201^. Amino acids Trp^43^, Trp^90^, Tyr^119^, and Trp^205^ in *S. meliloti* and Trp^40^ and Trp^87^ in *A. tumefaciens* are also important for the binding of choline (Oswald et al., 2008; Aktas et al., 2011), and the *B. suis* bv2 Thomsen homologs presented similar characteristics (Trp^47^, Trp^94^, Tyr^123^, and Trp^209^ for ChoX1; Trp^38^, Trp^86^, Tyr^109^, and Trp^199^ for ChoX2). Sequencing of the *choX1* and *choX2* orthologs showed that all the above-summarized characteristics of *B. suis* bv2 Thomsen were reproduced in Bs2WT. In contrast with ChoX1 and ChoX2, the BSUIS_A0222 encoded protein does not show any of the relevant features described in ChoX of *S. meliloti* and *A. tumefaciens*, in accordance with the results presented by Herrou et al. (2017) describing the *B. abortus* BSUIS_A0222 ortholog.

**FIGURE 3:**
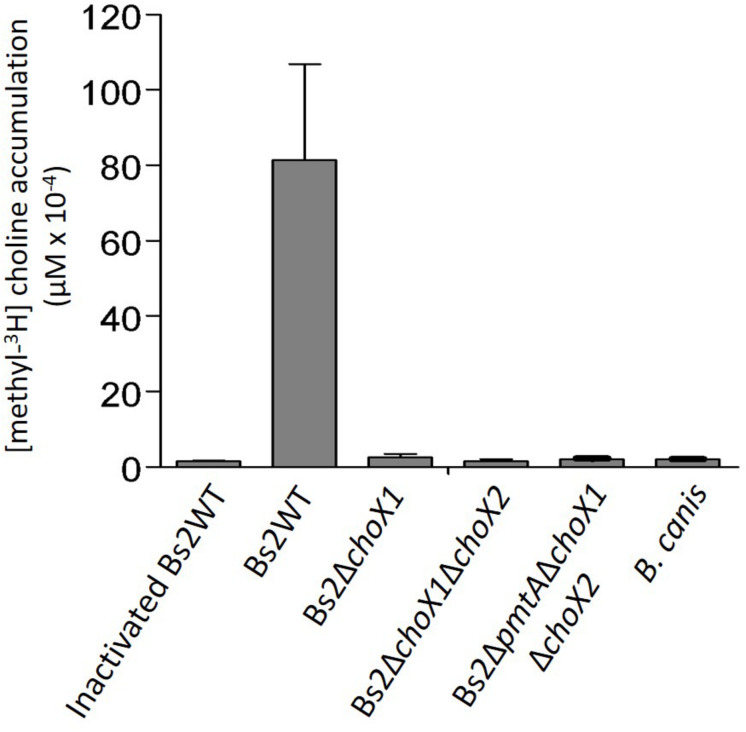
Choline accumulation in Bs2WT, Bs2Δ*choX1*, Bs2Δ*choX1*Δ*choX2*, Bs2Δ*pmtA*Δ*choX1*Δ*choX2*, and *B. canis* grown in mGSM. Values are the mean ± standard error of technical duplicates of a representative experiment, repeated at least two times with similar results.

We used the above-summarized data to investigate the importance of choline uptake in *pcs*-dependent PC synthesis in Bs2WT. First, we constructed a Bs2Δ*choX1* non-polar mutant. Also, since despite the absence of the Gly^120^–Ala^121^ and Asn^156^–Asp^157^ residues (described as relevant in *B. abortus*, Herrmann et al., 2013) ChoX2 still keeps features relevant in choline binding, we ensured removal of any residual activity by constructing a double Bs2Δ*choX1*Δ*choX2* mutant. Then, we grew the bacteria in mGSM supplemented with a low amount (0.04 μM) of methyl-^3^H-choline to avoid any interference of a potential low-affinity choline uptake system such as that described in *B. abortus* (Herrmann et al., 2013). Whereas viable Bs2WT accumulated [methyl-^3^H]-choline, neither Bs2Δ*choX1* nor Bs2Δ*choX*1Δ*choX*2 accumulated the probe either after 15 (Figure 3) or 60 min (not shown). Moreover, introduction of *choX*1 into Bs2Δ*choX1* restored the ability to accumulate [methyl-^3^H]-choline to the levels of Bs2WT (Supplementary Figure 6). These results are in keeping with the observations in *B. abortus* (Herrmann et al., 2013) and indicate that ChoXWV1 also provides the major pathway for choline import in Bs2WT.

### ChoX Is Not Conserved in All *Brucella* Species

Consistent with the postulated import of choline from the host Comerci et al. (2006); Herrmann et al. (2013) noted that the characteristics of a functional ChoX1 described in the previous paragraph are conserved in all *B. abortus* strains sequenced. However, when we extended the genomic analyses to other brucellae to confirm the association of ChoX1 conservation with parasitism, we observed two conspicuous exceptions: *B. canis* and *B. inopinata*. In all sequenced strains of *B. canis*, the *choX1* ortholog carries a deletion in the position equivalent to nucleotide 67 that creates a frameshift that extends to most of the gene (Supplementary Table 5). On the other hand, *choX2*, which is not related to choline uptake, is conserved (Supplementary Table 6). Strikingly, the same *choX1* frameshift observed in *B. canis* is also present in *B. inopinata* BO1. We did not find other remarkable mutations in ChoW1 and ChoV1 in either of these species (Supplementary Tables 7, 8). The presence of this *choX1* frameshift strongly suggests that wild-type *B. canis* and *B. inopinata* are unable to take up choline, at least under the experimental conditions used here. When we tested this prediction using *B. canis*, we found no accumulation of [methyl-^3^H]-choline (Figure 3).

### Phosphatidylcholine Synthesis in ChoX-Deficient Brucellae

**FIGURE 4:**
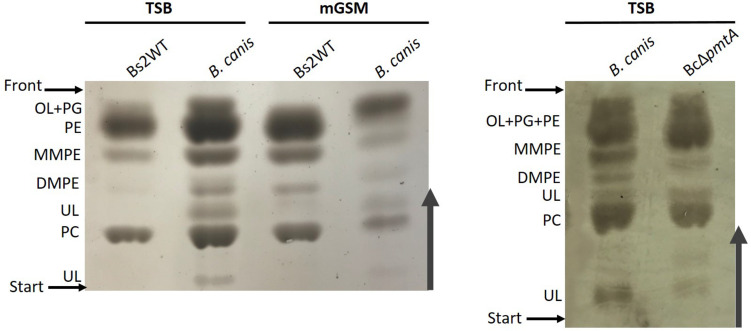
High-performance thin-layer chromatography analysis of the free lipids of *B. canis* and BcΔ*pmtA* grown in TSB or mGSM. OL, ornithine lipids; PG, phosphatidylglycerol; PE, phosphatidylethanolamine; MMPE, monomethyl-phosphatidylethanolamine; DMPE, dimethyl-phosphatidylethanolamine; PC, phosphatidylcholine; UL, unknown lipid.

*Brucella inopinata* is known to produce PC (Scholz et al., 2010), and according to one early report, *B. canis* also produces PC (Kulikov and Dranovskaia, 1988). Since both species are naturally ChoX deficient, it seems highly likely that these bacteria produce PC exclusively through the PmtA pathway and that Pcs, although conserved, no longer plays a role in PC synthesis. We tested this hypothesis by comparing the lipid profiles of wild-type *B. canis* and an isogenic *pmtA* mutant. We found that the parental strain produced PC in both TSB and mGSM (Figure 4), a result that shows that *B. canis* does not depend on the ChoXWV system to produce PC regardless of the complexity of the medium. However, we also observed that disabling *pmtA* in *B. canis* (BcΔ*pmtA* mutant) did not abrogate its capacity to synthesize PC in TSB (Figure 4).

**FIGURE 5:**
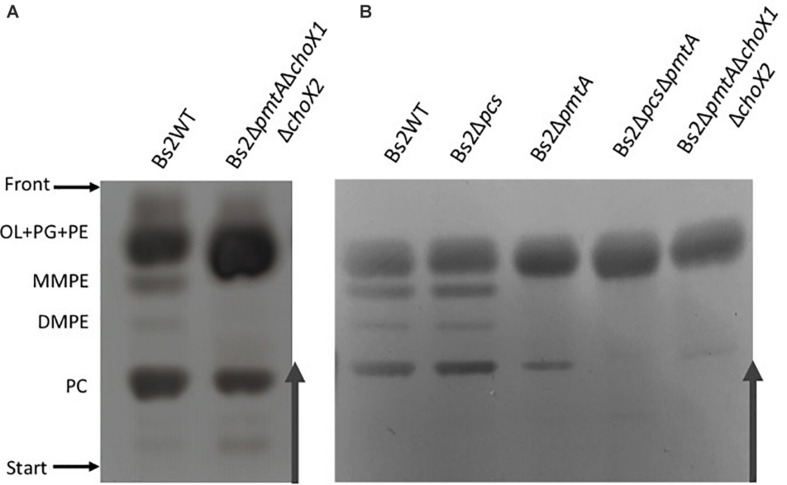
High-performance thin-layer chromatography analysis of the free lipids of Bs2WT- and Bs2-derived **(A)** mutants grown in TSB and **(B)** mutants grown in mGSM. OL, ornithine lipids; PG, phosphatidylglycerol; PE, phosphatidylethanolamine; MMPE, monomethyl-phosphatidylethanolamine; DMPE, dimethyl-phosphatidylethanolamine; PC, phosphatidylcholine; UL, unknown lipid.

**FIGURE 6:**
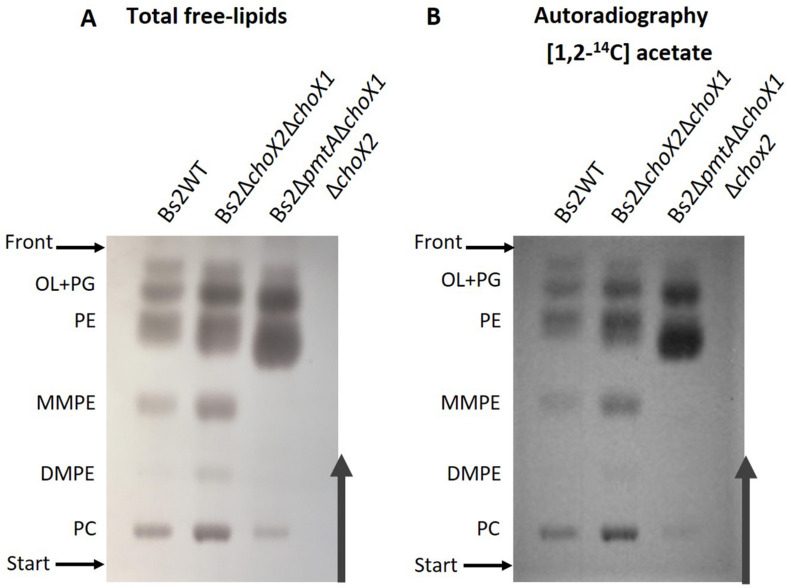
High-performance thin-layer chromatography analysis of Bs2WT, Bs2Δ*choX1*Δ*choX2*, and Bs2Δ*pmtA*Δ*choX1*Δ*choX2* after *in vivo* labeling with [1,2^− 14^C] acetate in mGSM. Total free lipids were revealed by charring **(A)** or autoradiography **(B)**. OL, ornithine lipids; PG, phosphatidylglycerol; PE, phosphatidylethanolamine; MMPE, monomethyl-phosphatidylethanolamine; DMPE, dimethyl-phosphatidylethanolamine; PC, phosphatidylcholine.

To further investigate PC synthesis by Pcs in ChoX-deficient brucellae, we carried out experiments in Bs2WT taking advantage of the mutants developed during this work. First, we combined the non-polar *pmtA* and *choX*1*choX2* mutations in a triple Bs2Δ*pmtA*Δ*choX1*Δ*choX2* mutant and, after confirming its inability to transport choline (Figure 3 and Supplementary Figure 7), we compared the free lipids produced by the parental strain and this mutant in TSB. While Bs2WT synthetized PC and the MMPE and DMPE precursors that confirm the activity of PmtA, Bs2Δ*pmtA*Δ*choX1*Δ*choX2* showed PC but not MMPE and DMPE (Figure 5A), a result that parallels that obtained with the BcΔ*pmtA* mutant. We then examined the free lipids of bacteria grown in mGSM. We first confirmed the functionality of the pathways in mGSM using Bs2Δ*pcs*, Bs2Δ*pmtA*, and a double Bs2Δ*pcs*Δ*pmtA* as controls. As expected, these mutants respectively showed MMPE, DMPE, and PC, reduced amounts of PC (as expected in a nutrient-limited medium), and no PC (Figure 5B). In mGSM, we detected traces of PC in Bs2Δ*pmtA*Δ*choX1*Δ*choX2* (Figure 5B; see also Figure 6A). When we extended these analyses to Bs2WT, Bs2Δ*choX1*Δ*choX2*, and Bs2Δ*pmtA*Δ*choX1*Δ*choX2* grown in [1,2^–14^C] acetate-mGSM, we also observed PC in Bs2WT and Bs2Δ*choX1*Δ*choX2* and traces of PC in Bs2Δ*pmtA*Δ*choX1*Δ*choX2* (Figure 6).

### Phosphatidylcholine Synthesis Is Required for Bs2WT Full Virulence in Mouse Model

**FIGURE 7:**
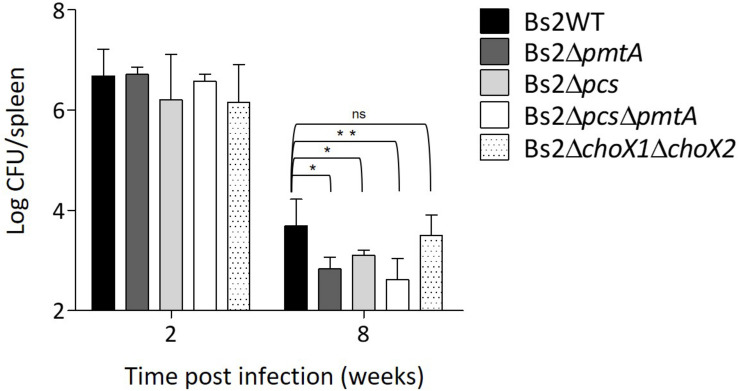
Phosphatidylcholine synthesis is required for Bs2WT full virulence. CFUs in spleen of infected BALB/c mice were counted after intraperitoneal inoculation with 10^5^ CFU/mouse of Bs2WT (parental strain), Bs2Δ*pcs*, Bs2Δ*pmtA*, Bs2Δ*pcs*Δ*pmtA*, or Bs2Δ*choX1*Δ*choX2*. *p ≤ 0.05, **p ≤ 0.01, n.s. not significant.

We used the mouse model (Grilló et al., 2012; Aragón-Aranda et al., 2020) to examine the contribution of PC to *B. suis* bv2 virulence. We observed that impairment of PC synthesis in Bs2WT by deletion of both *pcs* and *pmtA* resulted in a low degree of attenuation that only became significant at late stages of the infection (Figure 7). This observation is reminiscent of the attenuation of *B. abortus* 2308 PC-deficient mutants, which is also more marked at later stages of infection (Comerci et al., 2006; Conde-Álvarez et al., 2006). This profile was more clearly observed for the double mutant, suggesting that in Bs2WT, both pathways are active in this virulence model. However, when we tested Bs2Δ*choX1*Δ*choX2*, we did not observe an effect on virulence at any time (Figure 7), suggesting that PC synthesis in *B. suis* biovar 2 proceeds independently of ChoX *in vivo*.

## Discussion

Phosphatidylcholine has been shown to be required for the fitness of many α-2 *Proteobacteria* including *Brucella* and the successful interactions of these bacteria with their respective eukaryotic hosts (Comerci et al., 2006; Conde-Álvarez et al., 2006). Studies employing *B. abortus* 2308 and *B. melitensis* 16M suggested that the brucellae may rely exclusively on the Pcs pathway for PC biosynthesis based on the presence of dysfunctional *pmtA* genes (Comerci et al., 2006). However, considering the recently recognized physiologic and metabolic diversity that exists between *Brucella* strains (Scholz et al., 2008, 2010; Vizcaíno and Cloeckaert, 2012; Wattam et al., 2012; Whatmore et al., 2014; Soler-Lloréns et al., 2016; Barbier et al., 2017; Conde-Álvarez et al., 2018; Zúñiga-Ripa et al., 2018; Machelart et al., 2020; Lázaro-Antón et al., 2021), we thought it worthwhile to re-examine PC synthesis in these bacteria. We first investigated the functionality of the Pcs and PmtA pathways in a virulent strain of *B. suis* bv2, a choice based on both its position within the core brucellae (Whatmore, 2009) and biosafety considerations (this biovar is highly virulent in pigs but not in humans; Welfare, 2009). We found both pathways to be functional in this strain, and a subsequent genomic comparison across the genus showed that, in *Brucella*, there are polymorphisms in the metabolism of PC *via* PmtA. It is remarkable that the converse situation, e.g., Pcs dysfunction and PmtA functionality, does not occur in wild-type *Brucella* or in their close phylogenetic neighbors. For the latter, although the PmtA pathway may be more energetically demanding because it requires *S*-adenosyl-homocysteine recycling, it may be beneficial in the soil where competition for choline can occur. However, the Pcs pathway may be sufficient during the interactions of these bacteria with their eukaryotic hosts where choline should be available, which could explain why both pathways have been retained (Aktas et al., 2014a,b). Notably, the zoonotic brucellae do not multiply in nature outside their mammalian hosts, and the availability of host-derived choline has been proposed as the basis for the loss of functionality of the Pmt pathway in *B. abortus* and *B. melitensis* (Comerci et al., 2006). Why this has happened in these ruminant-associated strains but not in the other zoonotic *Brucella* species may reflect unknown characteristics of the nutritional environment in different animal hosts or the randomness that is at the basis of bacterial evolution.

Despite the proposed advantage of the Pcs pathway for *Brucella* strains in mammalian hosts, the corresponding choline transporter ChoXWV1 is not conserved in *B. canis* or *B. inopinata* BO1, and the results presented here show that the frameshift mutation present in the *B. canis choX1* gene inactivate high-affinity choline import in this strain. These studies also support those reported by Herrmann et al. (2013), which indicate that ChoXWV2 (unharmed in *B. canis*) is not involved in choline uptake, even though its conservation throughout the genus suggests that it may serve for the uptake of a substrate structurally akin to choline (Aragón-Aranda, unpublished observations). The ChoXWV1 dysfunction in *B. canis* was unexpected due to the close relatedness of this species to *B. suis* biovar 3 and 4 strains (Scholz et al., 2008; Whatmore, 2009; Wattam et al., 2014), which carry a canonical ChoX1. Finding the same mutation in the *B. inopinata* BO1 *choX1* gene was even more unexpected since this strain is phylogenetically very distant from *B. canis* and *B. suis* biovar 3 and 4 strains. While we do not know the reasons for this coincidence, these observations are not trivial because they pose questions on the origin of the choline used by the otherwise highly conserved Pcs pathway and on how it is obtained from the mammalian host.

Although unable to take up choline, *B. canis* keeps Pcs as well as PmtA and a BcΔ*pmtA* mutant maintains the ability to synthesize PC in TSB, a medium that contains choline (Figure 4). Similarly, Bs2Δ*pmtA*Δ*choX1*Δ*choX2* produced PC in this medium (Figure 5A). Moreover, even though definite proof of its identity requires a more refined analysis than HPTLC, Bs2Δ*pmtA* showed low amounts of a component compatible with PC but not MMPE or DMPE in nutrient-limited mGSM (Figure 5B). Low amounts of PC can also be observed in [1,2]^14^C-acetate autoradiograms of *B. abortus* 2308 and the isogenic BAB2_0502 (c*hoX2*)-BAB1_0226 (third *choX*) double mutant grown in Gerhardt’s synthetic medium (similar to mGSM) (Comerci et al., 2006; Herrmann et al., 2013). One possibility is that choline could be synthesized from ethanolamine by the action of non-previously described methylases and, although mGSM does not contain ethanolamine, ethanolamine could be derived from the action of phospholipases on PE *via* PLA-1 (Kerrinnes et al., 2015). However, while the results with radioisotopes and Bs2Δ*pmtA*Δ*choX1*Δ*choX2* studies (Figure 6 and Supplementary Figure 7) do not rigorously rule out this possibility, they show that such an endogenous source of choline is minimal in a nutrient-limited medium. An alternative explanation would be that Pmt-like enzymes produce PC in low amounts, thus making detection of MMPE and DMPE exceedingly difficult. In *Bradyrhizobium japonicum*, a PmtA homolog is required for efficient PC synthesis but PmtA mutants still produce minor amounts of PC (Minder et al., 2004). *B. japonicum* and *Bradyrhizobium* SEMIA 6144 R carry four and two additional Pmt enzymes, respectively (Aktas et al., 2010), and several *pmt* candidates exist in *Rhodopseudomonas palustris*, *Rhizobium etli*, and *R. leguminosarum* (Hacker et al., 2008). *Brucella* genomes also contain up to 11 additional genes encoding putative methyltransferases but cloning and expression of these genes in *E. coli* has failed to detect enzymatic activity consistent with a role in PC biosynthesis (Vences-Guzmán, unpublished observations). Finally, as TSB contains low amounts of choline, a plausible explanation for the presence of PC in Bs2Δ*pmtA*Δ*choX1*Δ*choX2* grown in this medium would be the activity of an alternative choline low-affinity uptake system, as proposed by Herrmann et al. (2013), and we favor this hypothesis. If such a system were relevant *in vivo*, e.g., in an environment with abundant choline, it could explain why the *pcs* gene is conserved in *Brucella*. However, what is clear is that the ChoXWV-Pcs and PmtA pathways are by far the major routes for PC biosynthesis in the genus *Brucella in vitro*. Nevertheless, there are dysfunctions that affect one pathway or the other in some *Brucella* species, and the differential use of these pathways may reflect important differences that exist between *Brucella* strains and/or the natural hosts they inhabit and whether they have an environmental niche outside of these natural hosts.

Previous evidence on the need for PC in fitness and virulence was obtained with *B. abortus* Pcs mutants in the same mouse model used here, and consistent with this, we observed that PC-lacking *B. suis* biovar 2 (i.e., BsΔ*pcs*Δ*pmtA*) displays similar defects. This effect was much less intense than that caused by mutations in the immunodominant lipopolysaccharide of *Brucella*, a molecule playing a chief role in escaping detection by innate immunity (Martirosyan et al., 2011). Therefore, it seems that the lack of PC has a comparatively small impact on the stealthiness of this parasite, which explains why it is manifested only in the chronic phase of infection in mice. It is also noteworthy that abrogation of high-affinity choline import in *B. suis* biovar 2 did not affect virulence under the same experimental conditions, suggesting that PC synthesis in *B. suis* biovar 2 *in vivo* proceeds independently of ChoX. This could also explain the existence of naturally ChoX-deficient virulent brucellae like *B. canis* and *B. inopinata*. Although *B. canis* presented low amounts of unknown lipids (Figure 4), we did not find that they could vary significantly upon growth in the two media or after *pmtA* mutation, evidence that could suggest membrane compensatory effects associated with PC deficiencies. It is worth noting that *B. inopinata* and other *Brucella* species produce low amounts of polar lipids that vary depending on the strain or species and whose identities and roles remain unknown (reviewed in Scholz et al., 2010). Interestingly, Herrmann et al. (2013) found that a *B. abortus* ChoX mutant was not attenuated 30 days after intragastric inoculation with 10^10^ CFU, which contrasts with the attenuation of an isogenic *B. abortus pcs* mutant using the same protocol. Indeed, it may be that choline availability during infection varies from the early to the late stages and depending on the route of inoculation and the laboratory model (Herrmann et al., 2013). In addition, Herrmann et al. (2013) also made the interesting observation that attenuation in macrophages was detected when Gerhardt’s synthetic medium but not when choline-rich TSB was used to prepare the inoculum. Therefore, whereas all authors conclude that PC is necessary for full virulence, it may be that different media for inoculum preparation and CFU counting, and different infection routes, yield similar but not identical results. The demonstration that most *Brucella* spp. keep the Pcs and the PmtA pathways and a functional ChoXWV, and that virulent *B. canis* also possesses both pathways but lacks a functional ChoXWV, opens the possibility of gaining further insight into the role of these three systems in *Brucella* virulence in natural hosts.

## Data Availability Statement

The original contributions presented in the study are included in the article/Supplementary Material, further inquiries can be directed to the corresponding author/s.

## Ethics Statement

The animal study was reviewed and approved by the procedures were in accordance with the current European (directive 86/609/EEC) and Spanish (RD 53/2013) legislations, supervised by both Ethical Committee for Animal Experimentation of CITA and Animal Welfare Committee of the University of Navarra and authorized by Aragón (reports No. 2014-20 and 2014-21) and Navarra (CEEA 045/12) governments.

## Author Contributions

RC-Á, MI, and IM conceived and coordinated the study. PM supervised the mouse studies. BA-A, LP-C, MS-B, MV-G, MM, LL-A, and AZ-R performed the experiments. RC-Á, BA-A, CS, and IM wrote the manuscript. All authors analyzed the results and approved the final version of the manuscript, and read and agreed to the published version of the manuscript.

## Conflict of Interest

The authors declare that the research was conducted in the absence of any commercial or financial relationships that could be construed as a potential conflict of interest.

## Publisher’s Note

All claims expressed in this article are solely those of the authors and do not necessarily represent those of their affiliated organizations, or those of the publisher, the editors and the reviewers. Any product that may be evaluated in this article, or claim that may be made by its manufacturer, is not guaranteed or endorsed by the publisher.
