# Supplementary material for: The Phospholipid N-Methyltransferase and Phosphatidylcholine Synthase Pathways and the ChoXWV Choline Uptake System Involved in Phosphatidylcholine Synthesis Are Widely Conserved in Most, but Not All Brucella Species
Source: Front Microbiol. 2021 Aug 4;12:614243. doi: 10.3389/fmicb.2021.614243 (PMC8371380; doi:10.3389/fmicb.2021.614243)
Supplement: Supplementary file 1 [file Data_Sheet_1.doc]

Supplementary Table S1. Bacterial strains and plasmids used

| **Strains** | **Characteristics** | **Reference** | |
| --- | --- | --- | --- |
| ***Brucella* spp.** |  |  | |
| Bs2WT | *B. suis* biovar 2 CITA 198; wild-type strain, smooth LPS | CITA collection | |
| Bs2Δ*pcs* | Bs2WT carrying an internal deletion in *pcs* (Δ_16-250_) | This work | |
| Bs2Δ*pmtA* | Bs2WT carrying an internal deletion in *pmtA* (Δ_35-168_) | This work | |
| Bs2Δ*pcs*Δ*pmtA* | Bs2WT carrying an internal deletion in *pcs* (Δ_16-250_) and in *pmtA* (Δ_35-168_) | This work | |
| Bs2Δ*pcs*Δ*pmtA_pcs* | Bs2Δ*pcs*Δ*pmtA* carrying pRCI-40 | This work | |
| Bs2Δ*pcs*Δ*pmtA_pmtA* | Bs2Δ*pcs*Δ*pmtA* carrying pBA-8 | This work | |
| Bs2Δ*choX1* | Bs2WT carrying an internal deletion in *choX1* (Δ_55-253_) | This work | |
| Bs2Δ*choX1_choX1* | Bs2Δ*choX*1 carrying pBA-13 | This work | |
| Bs2Δ*choX1*Δ*choX2* | Bs2WT carrying an internal deletion in *choX1* (Δ_55-253_) and in *choX2* (Δ_3-268_) | This work | |
| Bs2Δ*pmtA*Δ*choX1*  Δ*choX2* | Bs2WT carrying an internal deletion in *pmtA* (Δ_35-168_),  *choX1* (Δ_55-253_) and in *choX2* (Δ_3-268_) | This work | |
| *B. canis* | *B. canis* RM6/66 (ATCC23365); wild-type strain | Unav collection | |
| BcΔ*pmtA* | *B. canis* carrying an internal deletion in *pmtA* (Δ_35-168_) | This work | |
| ***Escherichia coli*** |  |  | |
| TOP10F´ | F-,lac/q Tn10 (Tetr) *mcr*A Δ(*mrr*-*hsd*RMS-*mcr*BC) 80l*ac*ZΔM15 Δ*lac*X74 *recA1ala*D139 Δ(*ara-leu*) 7697 *galU galK rspL endA1 nupG* | Invitrogen | |
| SM10 λpir | th-1 thr leu tonA lacY supE, recA::RP4-2-Tc::Mu KmR (λpir). | (Miller and Mekalanos, 1988) | |
| β2150 | F´lacZΔM15 *laclq* *pro*A+B+ *thr*B1004 *pro thi strA hsds ΔdapA::erm (Ermr) pir. E. coli* deficient in the diaminopimelic acid (DAP) synthesis | (Dehio and Meyer, 1997) | |
| S17 λ pir | Mating strain with plasmid RP4 inserted into the chromosome | (Simon et al., 1983) |  |
| PIR1 | F-Δlac169 rpoS(Am) robA1 creC510 hsdR514 endA recA1 uidA(ΔMluI)::pir-116 | Invitrogen |  |
| SM10 λpir | *th*-1 *thr* *leu* *tonA* *lacY* *supE*, *recA*::RP4-2-Tc::Mu KmR (λpir) | (Miller and Mekalanos 1988) |  |
| HB101 | F - *hsdS*20 *recA13* *ara*-14 *proA2* *lacY1* *galK2* *rpsL20* *xyl*-*5* *mtl*-1 *supE44* | (Sambrook, Fritsch, and Maniatis 1989) |  |
| **Plasmids** |  |  | |
| pRK2013 | Helper vector containing *tra* and *mob* genes | (Figurski and Helinski, 1979) | |
| pCR2.1 | Cloning vector, Km^R^ | Invitrogen | |
| pJQK | Derivative plasmid of pJQ200KS+; Km^R^; Gm^S^ | (Scupham and Triplett, 1997) | |
| pTNS2 | Plasmid expressing tnsABCD from Plac. ApR | (Choi et al. 2005) | |
| pUC18R6KTminiTn7Tkm | pUC18R6KTminiTn7T with Km cassette | (Llobet et al. 2009) | |
| pRCI-22 | *Bam*HI-*Xba*I fragment from pRCI-21 cloned into the corresponding site of pJQK | (Conde-Álvarez et al., 2006) | |
| pRCI-10 | *Bam*HI-*Xba*I fragment from pRCI-1 cloned into the corresponding site of pJQK | (Conde-Álvarez et al., 2006) | |
| pLPI-1 | 1263 bp of *B. abortus* chromosomal DNA containing the *choX1* deletion allele, generated by PCR and cloned into pCR2.1 | This work | |
| pLPI-2 | *Bam*HI-*Xba*I fragment from pLPI-1 cloned into the corresponding site of pJQK | This work | |
| pLPI-8 | 940 bp of *B. abortus* chromosomal DNA containing the *choX1* deletion allele, generated by PCR and cloned into pCR2.1 | This work | |
| pLPI-9 | *Bam*HI-*Xba*I fragment from pLPI-8 cloned into pJQK | This work | |
| pRCI-40 | *attL1-attL2* fragment of pDONOR-BMEII0695 cloned into the attR1-attR2 sites of pRH001 | (Conde-Álvarez et al., 2006) | |
| pRCI-41 | *attL1-attL2* fragment of pDONOR-BMEI2000 cloned into the attR1-attR2 sites of pRH001 | (Conde-Álvarez et al., 2006) | |
| pBA-8 | G62D *pmtA* version generated from pRCI-41 by PCR site-directed- mutagenesis | This work | |
| pBA-13 | *EcoR*I fragment of 1268 bp from Bs2WT chromosomal DNA containing the BSUIS_A1635 complete allele and its own promoter, cloned into the corresponding sites of pUC18 R6KT miniTn7T KmR. | This work | |

**Bibliography**

Conde-Álvarez, R., Grilló, M. J., Salcedo, S. P., de Miguel, M. J., Fugier, E., Gorvel, J. P., et al. (2006). Synthesis of phosphatidylcholine, a typical eukaryotic phospholipid, is necessary for full virulence of the intracellular bacterial parasite *Brucella abortus*. *Cell. Microbiol.* 8, 1322–1335. doi:10.1111/j.1462-5822.2006.00712.x.

Choi, Kyoung-Hee et al. 2005. “A Tn7-Based Broad-Range Bacterial Cloning and Expression System.” *Nature Methods* 2(6): 443–48.

Dehio, C., and Meyer, M. (1997). Maintenance of broad-host-range incompatibility group P and group Q plasmids and transposition of Tn5 in *Bartonella henselae* following conjugal plasmid transfer from *Escherichia coli*. *J. Bacteriol.* 179, 538–540. doi:10.1128/jb.179.2.538-540.1997.

Figurski, D. H., and Helinski, D. R. (1979). Replication of an origin-containing derivative of plasmid RK2 dependent on a plasmid function provided in trans. *Proc. Natl. Acad. Sci.* 76, 1648–1652. doi:10.1073/pnas.76.4.1648.

Llobet, Enrique, Catalina March, Paloma Giménez, and José A Bengoechea. 2009. *Klebsiella pneumoniae* OmpA Confers Resistance to Antimicrobial Peptides. *Antimicrobial Agents and Chemotherapy* 53(1): 298–302

Miller, V. L., and Mekalanos, J. J. (1988). A novel suicide vector and its use in construction of insertion mutations: osmoregulation of outer membrane proteins and virulence determinants in *Vibrio cholerae* requires toxR. *J. Bacteriol.*

Sambrook, J., E.F. Fritsch, and T Maniatis. 1989. *Molecular Cloning. A Laboratory Manual, 2nd Ed*. 2 nd. ed. Cold Spring Harbor Laboratory Press. New York: Cold Spring Harbor,

Scupham, A. J., and Triplett, E. W. (1997). Isolation and characterization of the UDP-glucose 4´-epimerase-encoding gene, *galE*, from *Brucella abortus* 2308. *Gene* 202, 53–59.

**Supplementary Table S2. Primers and PCR products expected in mutant construction and complementation**

| **Region amplified** | **Primers (sequence 5´-3´)** | **Amplified fragment (bp)** | | **Used for** | **Reference** |  |
| --- | --- | --- | --- | --- | --- | --- |
|  |  | **In the mutant** | **In the sibling revertant strain** |  |  |  |
| *pcs* | *pcs*-F1 (GAGCAGCACGGTATGGTAGC) | 981 | 1686 | Verifying the *pcs* deletion | (Conde-Álvarez et al., 2006) |  |
|  | *pcs*-R4 ( GCCGCATGAAATAAAATGGT) |  |  |  |  |  |
| *pcs* | *pcs*-F1 | --- | 795 | Verifying the *pcs* deletion | (Conde-Álvarez et al., 2006) |  |
|  | *pcs*-R5 (CGGAAATGAAGGACAGGTTC) |  |  |  |  |  |
| *pmtA* | *pmtA*-F1 (GGCTTCTTCTCCCGGATGAAGG) | 633 | 1035 | Verifying the *pmtA* deletion | (Conde-Álvarez et al., 2006) |  |
|  | *pmtA*-R4 (GCACGTCAAGGCCCACGATCAG) |  |  |  |  |  |
| *pmtA* | *pmtA-*F10 (CCGCTGTGCCCATGCTGAAT) | --- | 488 | Verifying the *pmtA* deletion | This work |  |
|  | *pmtA*-R4 |  |  |  |  |  |
| *choX1* | *choX1*-F1 (CGCTAAAAGCCTCGTCCA) | 493 | 493 | Overlapping PCR | This work |  |
|  | *choX1-*R2 (CCGTTGTCGAGGTGATGTC) |  |  |  |  |  |
| *choX1* | *choX1*-F3 (GACATCACCTCGACAACGGCCAATGTTGGCAAGTTCCTC) | 770 | 770 | Overlapping PCR | This work |  |
|  | *choX1*-R4 (ACCATGCCGATACCAAAGAA) |  |  |  |  |  |
| *choX1* | *choX1*-F1 | 1263 | 1854 | Verifying the *choX1* deletion | This work |  |
|  | *choX1*-R4 |  |  |  |  |  |
| *choX1* | *choX1*-F1 | --- | 673 | Verifying the *choX1* deletion | This work |  |
|  | *choX1*-R5 (CAGCGTCTCAACGGTCTTG) |  |  |  |  |  |
| *choX1* | *choX*1_Fw_Tn7 (5`- CCGGGCTGCAGGAATTTCATTTTCAACGGACGGGGT-3`) | 677 | 1268 | Complementation | This work |  |
|  | *choX*1_Rv_Tn7 (5`- AGCTTCTCGAGGAATTTCAGAGGCCGAGGGCCG-3`) |  |  |  |  |  |
| *choX2* | *choX2*-F1 (CAGGTTCATGCGGAATTTGT) | 446 | 446 | Overlapping PCR | This work |  |
|  | *choX2-*R2 (GCCCAACATACCTGCTCCT) |  |  |  |  |  |
| *choX2* | *choX2-*F3 (GGAGCAGGTATGTTGGGCAAAGGCCGTCGACAAATA) | 494 | 494 | Overlapping PCR | This work |  |
|  | *choX2-*R4 (TTTTCTACGCGGGCTACATC) |  |  |  |  |  |
| *choX2* | *choX2*-F1 | 940 | 1732 | Verifying the *choX2* deletion | This work |  |
|  | *choX2-*R4 |  |  |  |  |  |
| *choX2* | *choX2*-F1 | --- | 700 | Verifying the *choX2* deletion | This work |  |
|  | *choX2*-R5 (GGTAGCCACGCCATCATC) |  |  |  |  |  |
| Conde-Álvarez, R., Grilló, M. J., Salcedo, S. P., de Miguel, M. J., Fugier, E., Gorvel, J. P., et al. (2006). Synthesis of phosphatidylcholine, a typical eukaryotic phospholipid, is necessary for full virulence of the intracellular bacterial parasite *Brucella abortus*. *Cell. Microbiol.* 8, 1322–1335. doi:10.1111/j.1462-5822.2006.00712.x. | | | | | | |

**Supplementary Table S3. Comparison of amino acid sequences of PmtA of *Brucella* s****pp.**

| **Code** | **Strain** | **ORF** | | **Identity (%)** | **Length (aa)** | **Amino acid changes:** | |
| --- | --- | --- | --- | --- | --- | --- | --- |
|  |  |  |  |  |  | **outside the C.S.^1^** | **in the C.S.^.1^** |
| bmt | *Brucella suis* ATCC 23445 | | BSUIS_A1967 |  |  |  |  |
| bsui | *Brucella suis* bv. 2 Bs143CITA | | BSSP1_I1926 | 100 | 199 | --- | --- |
| bsuc | *Brucella suis* bv. 2 Bs364CITA | | BSSP2_I1931 | 100 | 199 | --- | --- |
| bsup | *Brucella suis* bv. 2 PT09143 | | BSPT1_I1942 | 100 | 199 | --- | --- |
| bsuv | *Brucella suis* bv. 2 PT09172 | | BSPT2_I1928 | 100 | 199 | --- | --- |
| bsz | *Brucella suis* bv. 3 | | DK67_243 | 100 | 199 | --- | --- |
| bmr | *Brucella microti* | | BMI_I2149 | 100 | 199 | --- | --- |
| bcar | *Brucella canis* RM6/66 | | DK60_58 | 100 | 199 | --- | --- |
| bcas | *Brucella canis* SVA13 | | DA85_10235 | 100 | 199 | --- | --- |
| bcs | *Brucella canis* ATCC 23365 | | BCAN_A2172 | 100 | 199 | --- | --- |
| bol | *Brucella canis* Oliveri | | BCOUA_I2127 | 100 | 199 | --- | --- |
| bsk | *Brucella canis* HSK A52141 | | BCA52141_I1794 | 100 | 199 | --- | --- |
| bvl | *Brucella vulpis* | | BF3285c1_0816 | 100 | 199 | --- | --- |
| bov | *Brucella ovis* | | BOV_2043 | 99 | 199 | K95E | --- |
| bsf | *Brucella suis* bv. 1 | | BSS2_I2061 | 99 | 199 | L164S | --- |
| bsi | *Brucella suis* 1330 | | BS1330_I2121 | 99 | 199 | L164S | --- |
| bms | *Brucella suis* 1330 | | BR2127 | 99 | 199 | L164S | L59-Dl^3^  G60R |
|  | *B. innopinata* BO1^2^ | |  | 100 | 199 |  |  |
|  | *Brucella* sp. NF 2653 (Austr.)^2^ | |  | 99 | 199 | V50A |  |
| bcet | *Brucella ceti* TE10759-12 | | V910_102017 | 99 | 199 | H92Q | --- |
| bpp | *Brucella pinnipedialis* B2/94 | | BPI_I2185 | 99 | 199 | G54D | --- |
| bme | *Brucella melitensis* bv. 1 16M | | BMEI2000 | 99 | 199 | --- | L59F |
| bmee | *Brucella melitensis* bv. 3 Ether | | DK62_1457 | 99 | 199 | --- | L59F |
| bmg | *Brucella melitensis* M5-90 | | BM590_A2117 | 99 | 199 | --- | L59F |
| bmi | *Brucella melitensis* ATCC 23457 | | BMEA_A2189 | 99 | 199 | --- | L59F |
| bmw | *Brucella melitensis* NI | | BMNI_I2028 | 99 | 199 | --- | L59F |
| bmz | *Brucella melitensis* M28 | | BM28_A2116 | 99 | 199 | --- | L59F |
| baa | *Brucella abortus* A13334 | | BAA13334_I00574 | 99 | 199 | --- | L59F; G62D |
| babc | *Brucella abortus* NCTC 10505 | | DO78_1964 | 99 | 199 | --- | L59F; G62D |
| bmc | *Brucella abortus* S19 | | BAbS19_I19930 | 99 | 199 | --- | L59F; G62D |
| bmf | *Brucella abortus* 2308 | | BAB1_2131 | 99 | 199 | --- | L59F; G62D |
| ^1.^C.S: Consensus sequence; PmtA SAM binding site [VL(E/D)XGXGXG]; ^2^ORF non available; ^3^Dl: deletion | | | | | | | |

**Supplementary Table S4. Comparison of amino acid sequences of Pcs of Brucella spp****.**

| **Code** | **Strain** | **ORF** | | **Identity (%)** | **Length (aa)** | **Amino acid changes:** | |
| --- | --- | --- | --- | --- | --- | --- | --- |
|  |  |  |  |  |  | **outside the C.S.^1^** | **in the C.S.^.1^** |
| bmt | *Brucella suis* ATCC 23445 | | BSUIS_B0568 |  |  |  |  |
| bsui | *Brucella suis* bv. 2 Bs143CITA | | BSSP1_II0527 | 100 | 266 | --- | --- |
| bsuc | *Brucella suis* bv. 2 Bs364CITA | | BSSP2_II0534 | 100 | 276 | --- | --- |
| bsup | *Brucella suis* bv. 2 PT09143 | | BSPT1_II0525 | 100 | 276 | --- | --- |
| bsuv | *Brucella suis* bv. 2 PT09172 | | BSPT2_II0528 | 100 | 276 | --- | --- |
| bsz | *Brucella suis* bv. 3 | | [DK67_2566](https://www.genome.jp/dbget-bin/www_bget?bsz:DK67_2566) | 100 | 266 | --- | --- |
| bcar | [*Brucella canis* RM6/66](https://www.genome.jp/dbget-bin/www_bfind?T03270) | | [DK60_2283](https://www.genome.jp/dbget-bin/www_bget?bcar:DK60_2283) | 100 | 266 | --- | --- |
| bcas | *Brucella canis* SVA13 | | DA85_13225 | 100 | 276 | --- | --- |
| bcs | [*Brucella suis* bv. 3](https://www.genome.jp/dbget-bin/www_bfind?T03266) | | [BCAN_B0572](https://www.genome.jp/dbget-bin/www_bget?bcs:BCAN_B0572) | 100 | 266 | --- | --- |
| bol | *Brucella canis* Oliveri | | BCOUA_II0572 | 100 | 276 | --- | --- |
| bsk | *Brucella canis* HSK A52141 | | BCA52141_II0369 | 100 | 276 | --- | --- |
| bsi | [*Brucella suis* 1330](https://www.genome.jp/dbget-bin/www_bfind?T02103) | | [BS1330_II0567](https://www.genome.jp/dbget-bin/www_bget?bsi:BS1330_II0567) | 100 | 266 | --- | --- |
| bms | *Brucella suis* 1330 | | BRA0572 | 100 | 266 | --- | --- |
|  | *B. innopinata* BO1^2^ | |  | 100 | 266 |  | --- |
|  | *Brucella* sp. NF 2653 (Austr.)^2^ | |  | 99 | 266 | M153T | --- |
| bvl | *Brucella vulpis* | | BF3285c2_0104 | 99 | 270 | M163T | --- |
| bov | [*Brucella ovis*](https://www.genome.jp/dbget-bin/www_bfind?T00534) | | [BOV_A0538](https://www.genome.jp/dbget-bin/www_bget?bov:BOV_A0538) | 99 | 270 | M153T | --- |
| bsf | [*Brucella suis* bv. 1](https://www.genome.jp/dbget-bin/www_bfind?T03181) | | [BSS2_II0544](https://www.genome.jp/dbget-bin/www_bget?bsf:BSS2_II0544) | 99 | 266 | M163T | --- |
| bmr | [*Brucella microti*](https://www.genome.jp/dbget-bin/www_bfind?T00955) | | [BMI_II566](https://www.genome.jp/dbget-bin/www_bget?bmr:BMI_II566) | 99 | 266 | M163T | --- |
| bcet | *Brucella ceti* TE10759-12 | | V910_200681 | 99 | 270 | M163T | --- |
| bpp | [*Brucella pinnipedialis* B2/94](https://www.genome.jp/dbget-bin/www_bfind?T01589) | | [BPI_II624](https://www.genome.jp/dbget-bin/www_bget?bpp:BPI_II624) | 99 | 266 | M163T | --- |
| bme | [*Brucella melitensis* bv. 1 16M](https://www.genome.jp/dbget-bin/www_bfind?T00072) | | [BMEII0695](https://www.genome.jp/dbget-bin/www_bget?bme:BMEII0695) | 99 | 266 | M163T | --- |
| bmee | [*Brucella melitensis* bv. 3 Ether](https://www.genome.jp/dbget-bin/www_bfind?T03393) | | [DK62_2861](https://www.genome.jp/dbget-bin/www_bget?bmee:DK62_2861) | 99 | 266 | M163T | --- |
| bmi | [*Brucella melitensis* ATCC 23457](https://www.genome.jp/dbget-bin/www_bfind?T00867) | | [BMEA_B0545](https://www.genome.jp/dbget-bin/www_bget?bmi:BMEA_B0545) | 99 | 266 | M163T | --- |
| bmw | [*Brucella melitensis* NI](https://www.genome.jp/dbget-bin/www_bfind?T01853) | | [BMNI_II0538](https://www.genome.jp/dbget-bin/www_bget?bmw:BMNI_II0538) | 99 | 270 | M163T | --- |
| babc | *Brucella abortus* NCTC 10505 | | DO78_2416 | 99 | 266 | M163T | --- |
| bmc | [*Brucella abortus* S19](https://www.genome.jp/dbget-bin/www_bfind?T00703) | | [BAbS19_II06250](https://www.genome.jp/dbget-bin/www_bget?bmc:BAbS19_II06250) | 99 | 266 | M163T | --- |
| bmf | [*Brucella abortus* 2308](https://www.genome.jp/dbget-bin/www_bfind?T00304) | | [BAB2_0668](https://www.genome.jp/dbget-bin/www_bget?bmf:BAB2_0668) | 99 | 266 | M163T | --- |
| bmg | *Brucella melitensis* M5-90 | | BM590_B0544 | 99 | 276 | R7M; M163T | --- |
| bmz | *Brucella melitensis* M28 | | BM28_B0544 | 99 | 276 | R7M; M163T | --- |
| baa | *Brucella abortus* A13334 | | BAA13334_II01275 | 98 | 276 | G3V;R7M; M163T | --- |
| ^1.^C.S: Consensus sequence of Pcs is [DG(X)2AR(X)8P(X)3G(X)3D(X)3D] [1]; ^2^ORF non available | | | | | | | |

**Supplementary Table S5.** **Comparison of amino acid sequences of ChoX1 of Brucella spp.**

| **Code** | **Strain** | **ORF** | **Identity (%)** | **Length (aa)** | **Amino acid change** |
| --- | --- | --- | --- | --- | --- |
| bmt | *Brucella suis* ATCC 23445 | BSUIS_A1635 |  |  |  |
| bsui | *Brucella suis* bv. 2 Bs134CITA | [BSSP1_I](https://www.kegg.jp/dbget-bin/www_bget?bsuc:BSSP2_I1600)1597 | 100 | 322 | --- |
| bsuc | *Brucella suis* bv. 2 Bs364CITA | [BSSP2_I1600](https://www.kegg.jp/dbget-bin/www_bget?bsuc:BSSP2_I1600) | 100 | 322 | --- |
| bsup | *Brucella suis* bv. 2 PT09143 | BSPT1_I1613 | 100 | 322 | --- |
| bsuv | *Brucella suis* bv. 2 PT09172 | [BSPT2_I1595](https://www.kegg.jp/dbget-bin/www_bget?bsuv:BSPT2_I1595) | 100 | 322 | --- |
| bsz | *Brucella suis* bv. 3 | DK67_747 | 98 | 322 | V4I; D14G;  M267I; A278D |
| bmr | *Brucella microti* | BMI_I1592 | 99 | 322 | D14G; A278D |
| bcar | *Brucella canis* RM6/66 | DK60_1590 | 98 | 248 | Frameshift (67 nt)  M267I; A278D |
| bcas | *Brucella canis* SVA13 | DA85_07575  Pseudogene | 98 | 248 | Frameshift (67 nt)  M267I; A278D |
| bcs | *Brucella canis* ATCC 23365 | BCAN_A1614 | 98 | 248 | Frameshift (67 nt)  M267I; A278D |
| bol | *Brucella canis* Oliveri | No found |  |  |  |
| bsk | *Brucella canis* HSK A52141 | BCA52141_I2660 | 98 | 248 | Frameshift (67 nt)  M267I; A278D |
| bvl | *Brucella vulpis* | BF3285c1_1383 | 98 | 322 | D14G; D88N;  A278D; R217K |
| bov | *Brucella ovis* | BOV_1524 | 99 | 322 | D14G; A278D |
| bsf | *Brucella suis* bv. 1 | BSS2_I1532 | 99 | 322 | D14G; M267I;  A278D |
| bsi | *Brucella suis* 1330 | BS1330_I1573 | 99 | 322 | D14G; M267I;  A278D |
| bms | *Brucella suis* 1330 | BR1579 | 99 | 322 | D14G; M267I;  A278D |
|  | *B. innopinata* BO1^1^ |  | 98 | 248 | Frameshift (67 nt)  M267I; A278D |
|  | *Brucella* sp. NF 2653 (Austr.)^1^ |  | 99 | 322 | D15G; A278D |
| bcet | *Brucella ceti* TE10759-12 | V910_100437 | 99 | 322 | D14G; A278D |
| bpp | *Brucella pinnipedialis* B2/94 | BPI_I1632 | 99 | 322 | D14G; A278D |
| bme | *Brucella melitensis* bv. 1 16M | BMEI0441 | 99 | 326 | D14G; A278D |
| bmee | *Brucella melitensis* bv. 3 Ether | DK62_1964 | 99 | 322 | D14G  A278D |
| bmg | *Brucella melitensis* M5-90 | BM590_A1571 | 98 | 322 | D14G; L82V;  E202D; A278D |
| bmi | *Brucella melitensis* ATCC 23457 | BMEA_A1632 | 98 | 322 | D14G; L82V;  E202D; A278D |
| bmw | *Brucella melitensis* NI | BMNI_I1520 | 98 | 322 | D14G; L82V;  E202D; A278D |
| bmz | *Brucella melitensis* M28 | BM28_A1585 | 98 | 322 | D14G; L82V;  E202D; A278D |
| baa | *Brucella abortus* A13334 | BAA13334_I01448 | 99 | 322 | D14G; A278D; S318L |
| babc | *Brucella abortus* NCTC 10505 | DO78_1451 | 99 | 322 | D14G; A278D;  S318L |
| bmc | *Brucella abortus* S19 | BAbS19_I14910 | 99 | 322 | D14G; A278D;  S318L |
| bmf | *Brucella abortus* 2308 | BAB1_1593 | 99 | 322 | D14G; A278D;  S318L |
| ^1^ORF non available | | |  |  |  |

**Supplementary Table S6. Comparison of amino acid sequences of ChoX2 of Brucella spp.**

| **Code** | **Strain** | **ORF** | **Identity**  **(%)** | **Length (aa)** | **Amino acid change** |
| --- | --- | --- | --- | --- | --- |
| bmt | *Brucella suis* ATCC 23445 | BSUIS_B0730 |  |  |  |
| bsui | *Brucella suis* bv. 2 Bs134CITA | BSSP1_II0678 | 100 | 288 | --- |
| bsuc | *Brucella suis* bv. 2 Bs364CITA | BSSP2_II0689 | 100 | 288 | --- |
| bsup | *Brucella suis* bv. 2 PT09143 | BSPT1_II0676 | 100 | 288 | --- |
| bsuv | *Brucella suis* bv. 2 PT09172 | BSPT2_II0678 | 100 | 288 | --- |
| bsz | *Brucella suis* bv. 3 | DK67_2700 | 100 | 288 | --- |
| bmr | *Brucella microti* | BMI_II731 | 100 | 288 | --- |
| bcar | *Brucella canis* RM6/66 | DK60_2435 | 100 | 288 | --- |
| bcas | *Brucella canis* SVA13 | DA85_13995 | 100 | 288 | --- |
| bcs | *Brucella canis* ATCC 23365 | BCAN_B0746 | 100 | 288 | --- |
| bol | *Brucella canis* Oliveri | BCOUA_II0738 | 100 | 288 | --- |
| bsk | *Brucella canis* HSK A52141 | BCA52141_II0166 | 100 | 288 | --- |
| bvl | *Brucella vulpis* | BF3285c2_0271 | 100 | 288 | --- |
| bov | *Brucella ovis* | Not found |  |  |  |
| bsf | *Brucella suis* bv. 1 | BSS2_II0701 | 100 | 288 | --- |
| bsi | *Brucella suis* 1330 | BS1330_II0731 | 100 | 288 | --- |
| bms | *Brucella suis* 1330 | BRA0738 | 100 | 288 |  |
|  | *B. innopinata* BO1^1^ |  |  |  | Frameshift  (222 nt) |
|  | *Brucella* sp. NF 2653 (Austr.)^1^ |  | 100 | 288 |  |
| bcet | *Brucella ceti* TE10759-12 | Not found |  |  |  |
| bpp | *Brucella pinnipedialis* B2/94 | BPI_II791 | 100 | 288 | --- |
| bme | *Brucella melitensis* bv. 1 16M | BMEII0550 | 99.7 | 288 | I127V |
| bmee | *Brucella melitensis* bv. 3 Ether | DK62_2706 | 99.3 | 288 | M84I; I127V |
| bmg | *Brucella melitensis* M5-90 | BM590_B0703 | 99.7 | 288 | I127V |
| bmi | *Brucella melitensis* ATCC 23457 | BMEA_B0716 | 99.7 | 288 | I127V |
| bmw | *Brucella melitensis* NI | BMNI_II0686 | 99.7 | 288 | I127V |
| bmz | *Brucella melitensis* M28 | BM28_B0705 | 99.7 | 288 | I127V |
| baa | *Brucella abortus* A13334 | BAA13334_II01017 | 99.3 | 288 | I127V; N238D |
| babc | *Brucella abortus* NCTC 10505 | DO78_2571 | 99 | 288 | G34D; I127V;  N238D |
| bmc | *Brucella abortus* S19 | BAbS19_II04730 | 99.3 | 288 | I127V; N238D |
| bmf | *Brucella abortus* 2308 | BAB2_0502 | 99.3 | 288 | I127V; N238D |
| ^1^ORF non available | | | | | |
|  |  |  |  |  |  |
|  |  |  |  |  |  |

**Supplementary Table S7. Comparison of amino acid sequences of ChoW1 of Brucella spp.**

| **Code** | **Strain** | **ORF** | **Identity (%)** | **Length (aa)** | **Amino acid changes** |
| --- | --- | --- | --- | --- | --- |
| bmt | *Brucella suis* ATCC 23445 | BSUIS_A1636 |  |  |  |
| bsui | *Brucella suis* bv. 2 Bs143CITA | BSSP1_I1598 | 100 | 278 | --- |
| bsuc | *Brucella suis* bv. 2 Bs364CITA | BSSP2_I1601 | 100 | 278 | --- |
| bsup | *Brucella suis* bv. 2 PT09143 | BSPT1_I1614 | 100 | 278 | --- |
| bsuv | *Brucella suis* bv. 2 PT09172 | BST2_I1596 | 100 | 278 | --- |
| bsz | *Brucella suis* bv. 3 | DK67_746 | 99 | 278 | L17S; G43D |
| bmr | *Brucella microti* | BMI_I1593 | 99 | 278 | L17S |
| bcar | *Brucella canis* RM6/66 | DK60_1591 | 99 | 278 | L17S; G43D |
| bcas | *Brucella canis* SVA13 | DA85_07580 | 99 | 278 | L17S; G43D |
| bcs | *Brucella canis* ATCC 23365 | BCAN_A1615 | 99 | 278 | L17S; G43D |
| bol | *Brucella canis* Oliveri | BCOUA_I1580 | 99 | 278 | L17S; G43D |
| bsk | *Brucella canis* HSK A52141 | BCA52141_I2658 | 99 | 278 | L17S; G43D |
| bvl | *Brucella vulpis* | BF3285c1_1382 | 99 | 278 | L17S; I57N; G88S |
| bov | *Brucella ovis* | BOV_1525 | 99 | 278 | L17S |
| bsf | *Brucella suis* bv. 1 | BSS2I1533 | 99 | 278 | L17S; G43D |
| bsi | *Brucella suis* 1330 | BS1330_I1574 | 99 | 278 | L17S; G43D |
| bms | *Brucella suis* 1330 | BR1580 | 99 | 278 | L17S; G43D |
|  | *B. innopinata* BO1^1^ |  | 99 | 278 | L17S;G43D |
|  | *Brucella* sp. NF 2653 (Austr.)^1^ |  | 99 | 278 | L54F;A118V |
| bcet | *Brucella ceti* TE10759-12 | V910_100436 | 99 | 278 | L17S |
| bpp | *Brucella pinnipedialis* B2/94 | BPI_I1633 | 99 | 278 | L17S |
| bme | *Brucella melitensis* bv. 1 16M | BMEI0440 | 99 | 278 | L17S; G177S |
| bmee | *Brucella melitensis* bv. 3 Ether | DK62_1963 | 99 | 278 | L17S; G177S |
| bmg | *Brucella melitensis* M5-90 | BM590_A1572 | 99 | 278 | L17S; G177S |
| bmi | *Brucella melitensis* ATCC 23457 | BMEA_A1633 | 99 | 278 | L17S; G177S |
| bmw | *Brucella melitensis* NI | BMNI_I1521 | 99 | 278 | L17S; G177S |
| bmz | *Brucella melitensis* M28 | BM28_A1586 | 99 | 278 | L17S; G177S |
| baa | *Brucella abortus* A13334 | BAA13334_I01447 | 99 | 278 | L17S: G177S; A194T |
| babc | *Brucella abortus* NCTC 10505 | DO78_1452 | 99 | 278 | L17S; P157L; G177S |
| bmc | *Brucella abortus* S19 | BAbS19_I14920 | 99 | 278 | L17S: G177S; A194T |
| bmf | *Brucella abortus* 2308 | BAB1_1594 | 99 | 278 | L17S: G177S; A194T |
| ^1^ORF non available | |  |  |  |  |

**Supplementary Table S8. Comparison of amino acid sequences of ChoV1 of Brucella spp.**

| **Code** | **Strain** | **ORF** | **Identity (%)** | | **Length (aa)** | | **Amino acid changes** |
| --- | --- | --- | --- | --- | --- | --- | --- |
| bmt | *Brucella suis* ATCC 23445 | BSUIS_A1637 | |  | |  |  |
| bsui | *Brucella suis* bv. 2 Bs143CITA | BSSP1_I1599 | | 99 | | 348 | Q273P |
| bsuc | *Brucella suis* bv. 2 Bs364CITA | BSSP2_I1602 | | 99 | | 348 | Q273P |
| bsup | *Brucella suis* bv. 2 PT09143 | BSPT1_I1615 | | 99 | | 348 | Q273P |
| bsuv | *Brucella suis* bv. 2 PT09172 | BSPT2_I1597 | | 100 | | 348 | --- |
| bsz | *Brucella suis* bv. 3 | DK67_745 | | 99 | | 348 | G138D; Q273P |
| bmr | *Brucella microti* | BMI_I1594 | | 99 | | 348 | D42G; Q273P |
| bcar | *Brucella canis* RM6/66 | DK60_1592 | | 99 | | 348 | G138D; Q273P |
| bcas | *Brucella canis* SVA13 | DA85_07585 | | 99 | | 348 | G138D; Q273P |
| bcs | *Brucella canis* ATCC 23365 | BCAN_A1616 | | 99 | | 348 | G138D; Q273P |
| bol | *Brucella canis* Oliveri | BCOUA_I1581 | | 99 | | 348 | G138D; Q273P |
| bsk | *Brucella canis* HSK A52141 | BCA52141_I2656 | | 99 | | 348 | G138D; Q273P |
| bvl | *Brucella vulpis* | BF3285c1_1381 | | 99 | | 348 | D42G; Q273P |
| bov | *Brucella ovis* | BOV_1526 | | 99 | | 348 | D42G; Q273P |
| bsf | *Brucella suis* bv. 1 | BSS2_I1534 | | 99 | | 348 | Q273P |
| bsi | *Brucella suis* 1330 | BS1330_I1575 | | 99 | | 348 | Q273P |
| bms | *Brucella suis* 1330 | BR1581 | | 99 | | 348 | Q273P |
|  | *B. innopinata* BO1^1^ |  | |  | |  | G138D; Q273P |
|  | *Brucella* sp. NF 2653 (Austr.)^1^ |  | |  | |  | 5 changes |
| bcet | *Brucella ceti* TE10759-12 | V910_100435 | | 99 | | 348 | D42G; Q273P |
| bpp | *Brucella pinnipedialis* B2/94 | BPI_I1634 | | 99 | | 348 | D42G; Q273P |
| bme | *Brucella melitensis* bv. 1 16M | BMEI0439 | | 99 | | 348 | D42G; Q273P |
| bmee | *Brucella melitensis* bv. 3 Ether | DK62_1962 | | 99 | | 348 | D42G; Q273P |
| bmg | *Brucella melitensis* M5-90 | BM590_A1573 | | 99 | | 348 | D42G; Q273P |
| bmi | *Brucella melitensis* ATCC 23457 | BMEA_A1634 | | 99 | | 348 | D42G; Q273P |
| bmw | *Brucella melitensis* NI | BMNI_I1522 | | 99 | | 348 | D42G; Q273P |
| bmz | *Brucella melitensis* M28 | BM28_A1587 | | 99 | | 348 | D42G; Q273P |
| baa | *Brucella abortus* A13334 | BAA13334_I01445 | | 99 | | 348 | D42G; Q273P |
| babc | *Brucella abortus* NCTC 10505 | DO78_1453 | | 99 | | 348 | D42G; Q273P |
| bmc | *Brucella abortus* S19 | BAbS19_I14930 | | 99 | | 348 | D42G; Q273P |
| bmf | *Brucella abortus* 2308 | BAB1_1595 | | 99 | | 348 | D42G; Q273P |
| ^1^ORF non available | |  | |  | |  |  |
