# Supplementary material for: The Phospholipid N-Methyltransferase and Phosphatidylcholine Synthase Pathways and the ChoXWV Choline Uptake System Involved in Phosphatidylcholine Synthesis Are Widely Conserved in Most, but Not All Brucella Species
Source: Front Microbiol. 2021 Aug 4;12:614243. doi: 10.3389/fmicb.2021.614243 (PMC8371380; doi:10.3389/fmicb.2021.614243)
Supplement: Supplementary file 2 [file Presentation_1.pptx]

## Slide 1
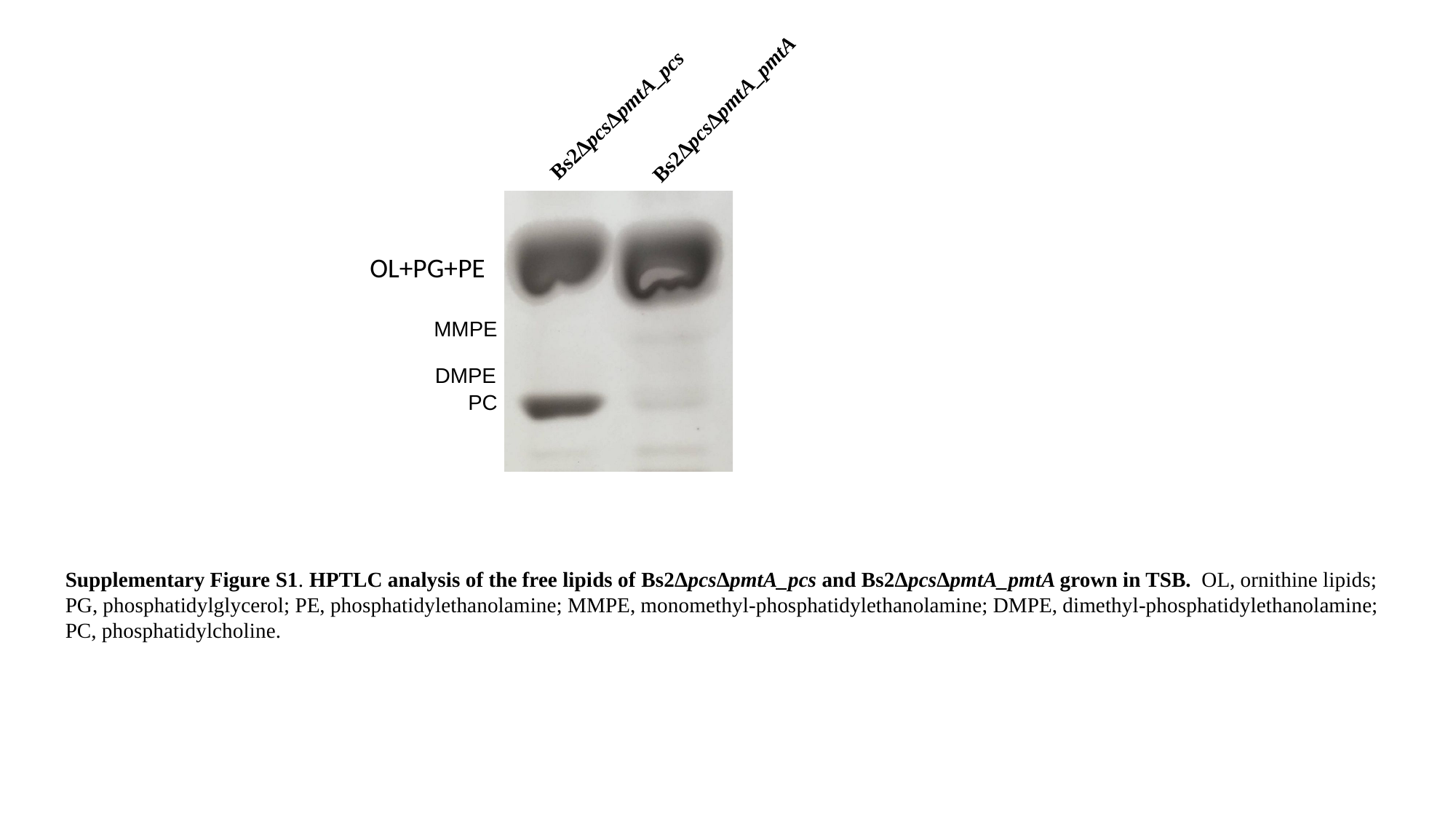

Bs2ΔpcsΔpmtA_pmtA
Bs2ΔpcsΔpmtA_pcs
OL+PG+PE
MMPE
DMPE
PC
Supplementary Figure S1. HPTLC analysis of the free lipids of Bs2ΔpcsΔpmtA_pcs and Bs2ΔpcsΔpmtA_pmtA grown in TSB. OL, ornithine lipids; PG, phosphatidylglycerol; PE, phosphatidylethanolamine; MMPE, monomethyl-phosphatidylethanolamine; DMPE, dimethyl-phosphatidylethanolamine; PC, phosphatidylcholine.

## Slide 2
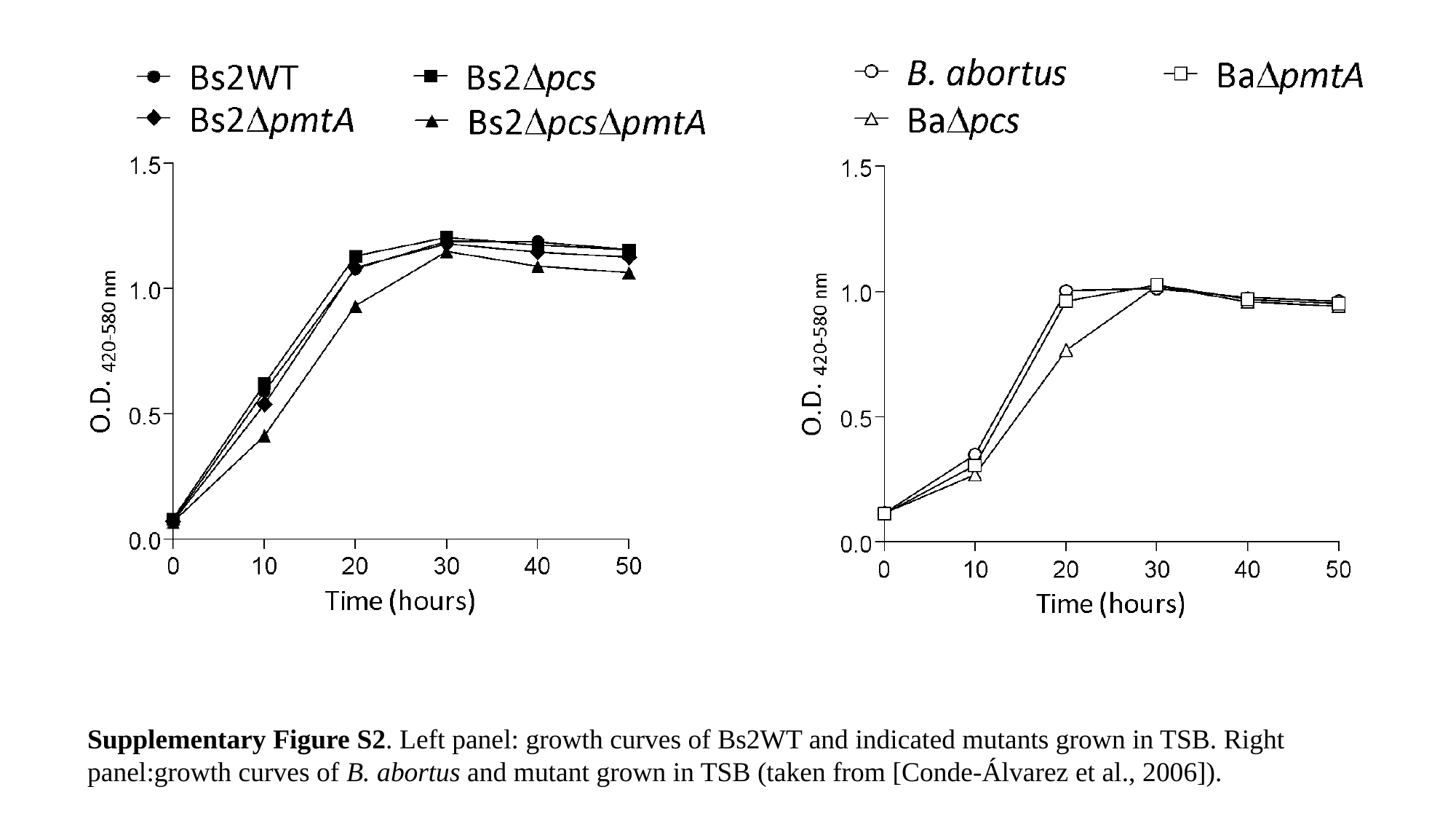

Supplementary Figure S2. Left panel: growth curves of Bs2WT and indicated mutants grown in TSB. Right panel:growth curves of B. abortus and mutant grown in TSB (taken from [Conde-Álvarez et al., 2006]).

## Slide 3
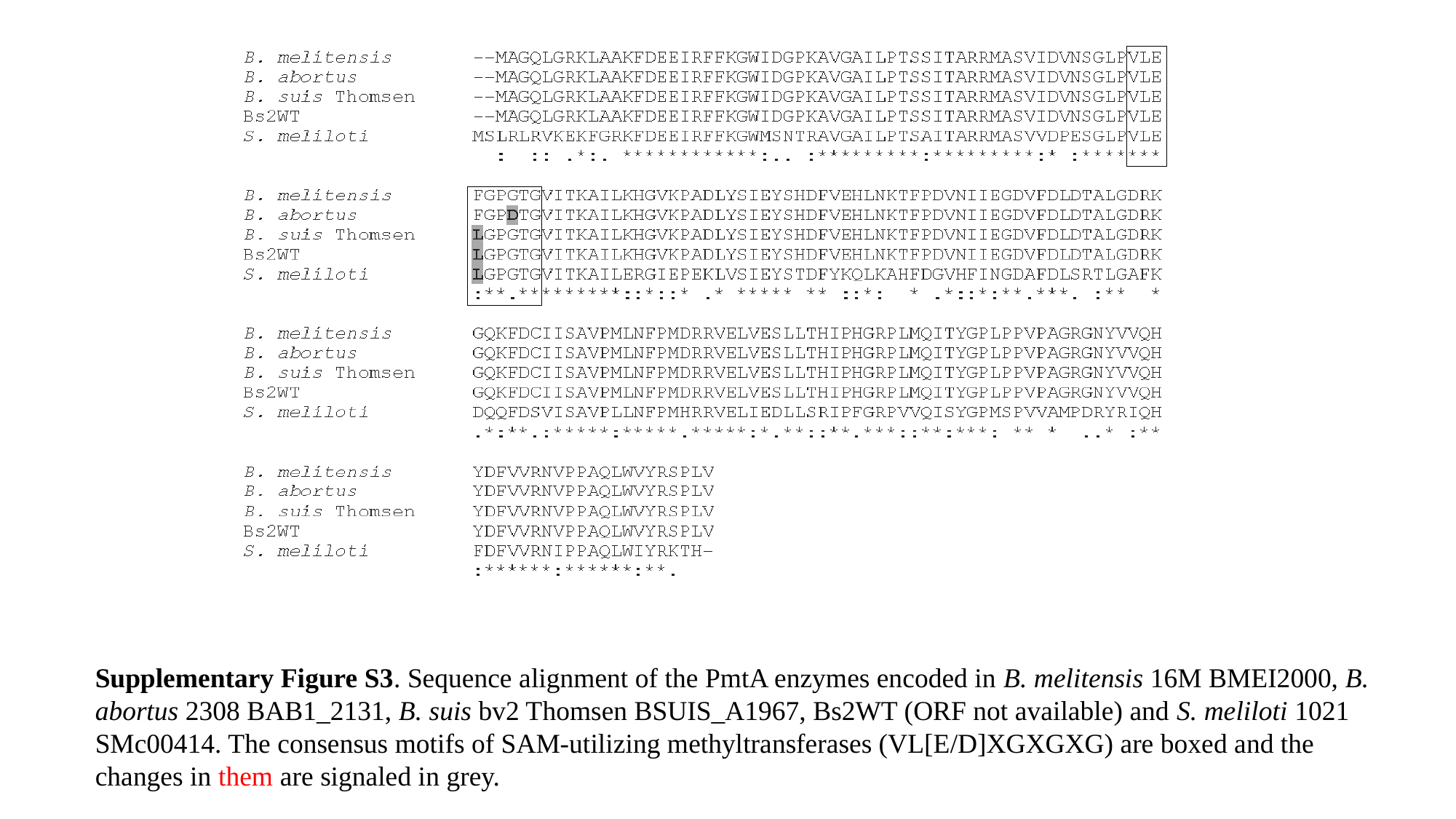

Supplementary Figure S3. Sequence alignment of the PmtA enzymes encoded in B. melitensis 16M BMEI2000, B. abortus 2308 BAB1_2131, B. suis bv2 Thomsen BSUIS_A1967, Bs2WT (ORF not available) and S. meliloti 1021 SMc00414. The consensus motifs of SAM-utilizing methyltransferases (VL[E/D]XGXGXG) are boxed and the changes in them are signaled in grey.

## Slide 4
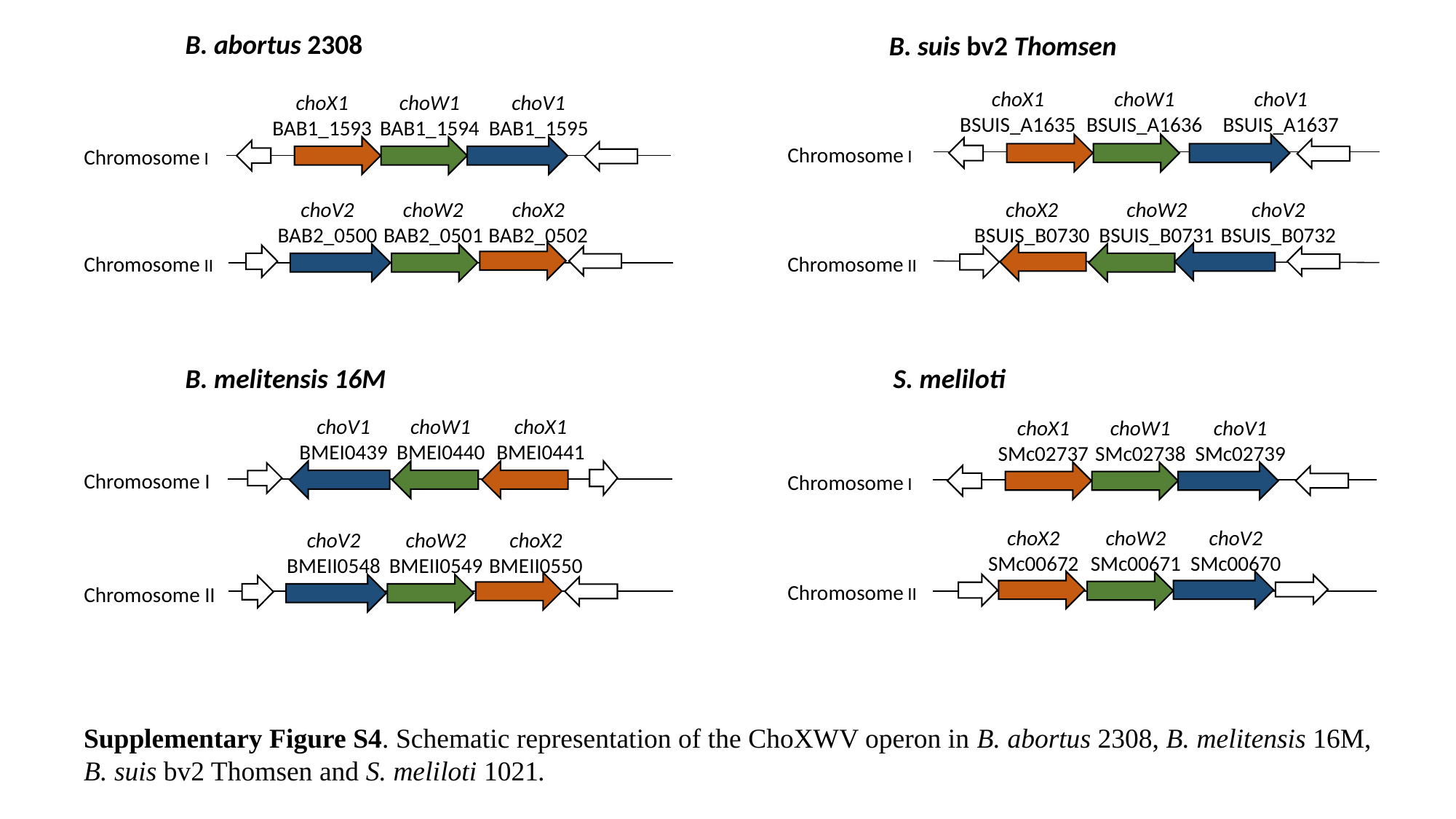

B. abortus 2308
B. suis bv2 Thomsen
choX1
BSUIS_A1635
choW1
BSUIS_A1636
choV1
BSUIS_A1637
choX1
BAB1_1593
choW1
BAB1_1594
choV1
BAB1_1595
Chromosome I
Chromosome I
choX2
BSUIS_B0730
choW2
BSUIS_B0731
choV2
BSUIS_B0732
choV2
BAB2_0500
choW2
BAB2_0501
choX2
BAB2_0502
Chromosome II
Chromosome II
B. melitensis 16M
S. meliloti
choV1
BMEI0439
choW1
BMEI0440
choX1
BMEI0441
choX1
SMc02737
choW1
SMc02738
choV1
SMc02739
Chromosome I
Chromosome I
choX2
SMc00672
choW2
SMc00671
choV2
SMc00670
choV2
BMEII0548
choW2
BMEII0549
choX2
BMEII0550
Chromosome II
Chromosome II
Supplementary Figure S4. Schematic representation of the ChoXWV operon in B. abortus 2308, B. melitensis 16M, B. suis bv2 Thomsen and S. meliloti 1021.

## Slide 5
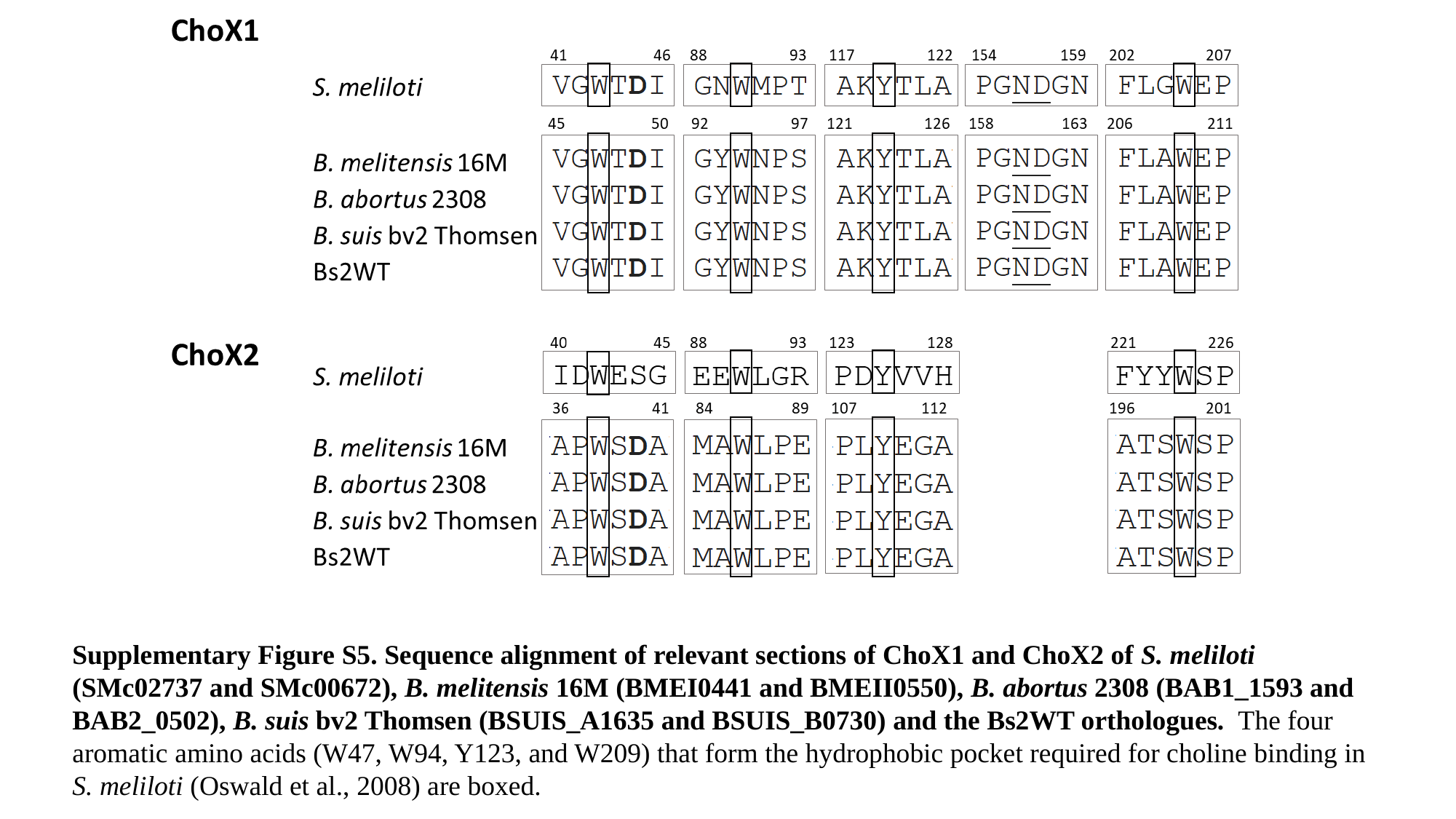

Supplementary Figure S5. Sequence alignment of relevant sections of ChoX1 and ChoX2 of S. meliloti (SMc02737 and SMc00672), B. melitensis 16M (BMEI0441 and BMEII0550), B. abortus 2308 (BAB1_1593 and BAB2_0502), B. suis bv2 Thomsen (BSUIS_A1635 and BSUIS_B0730) and the Bs2WT orthologues. The four aromatic amino acids (W47, W94, Y123, and W209) that form the hydrophobic pocket required for choline binding in S. meliloti (Oswald et al., 2008) are boxed.

## Slide 6
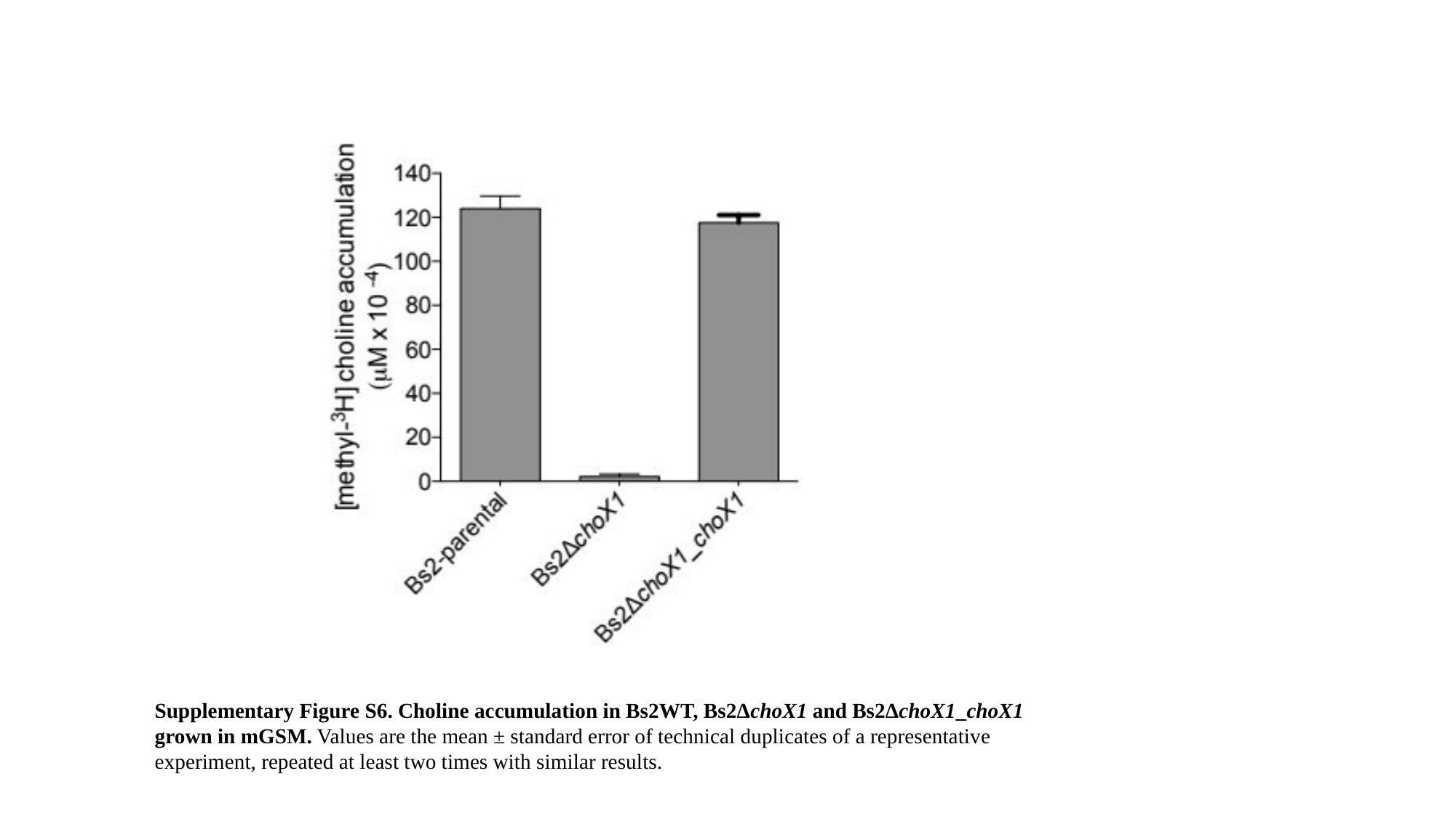

Supplementary Figure S6. Choline accumulation in Bs2WT, Bs2ΔchoX1 and Bs2ΔchoX1_choX1 grown in mGSM. Values are the mean ± standard error of technical duplicates of a representative experiment, repeated at least two times with similar results.

## Slide 7
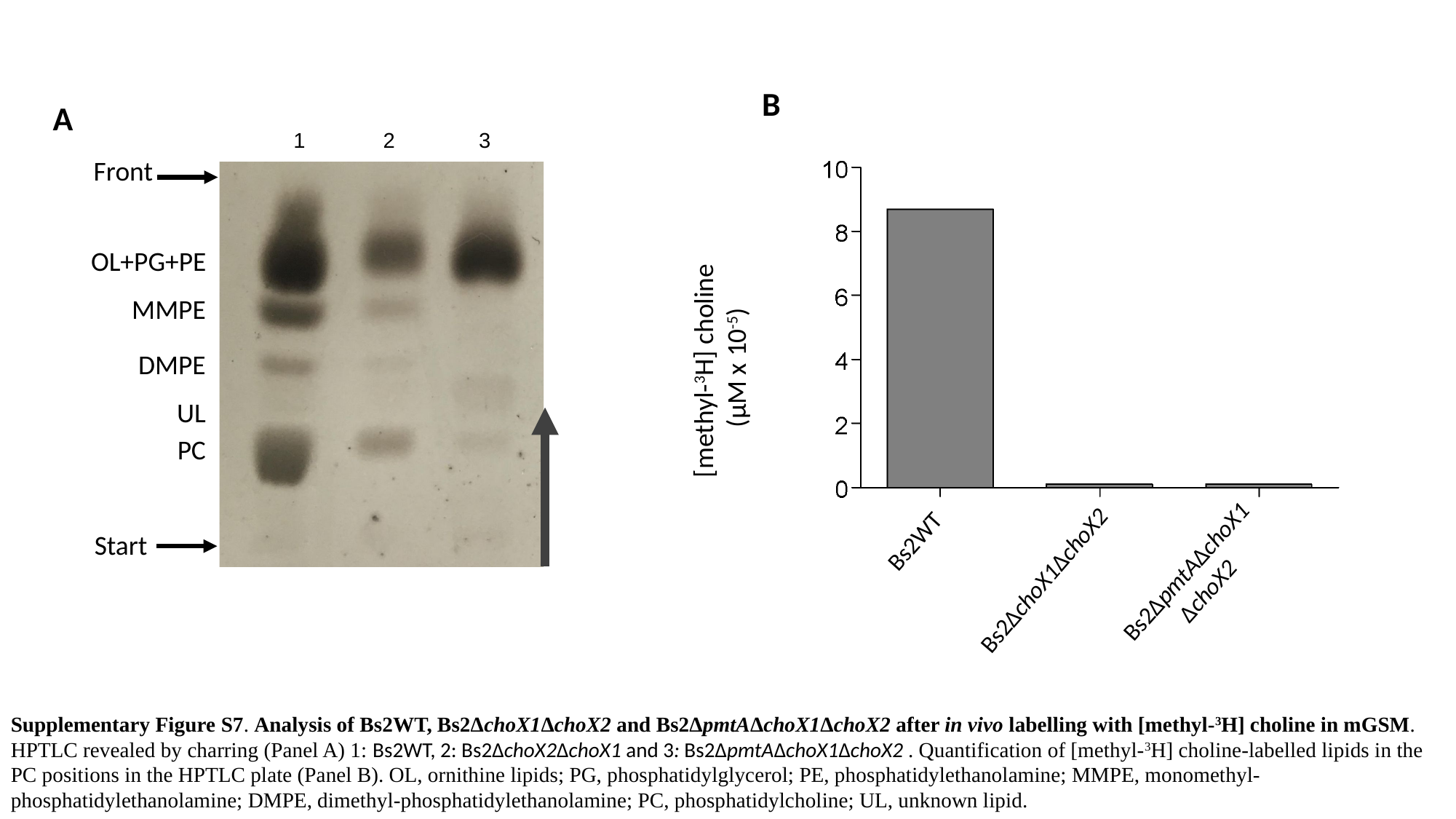

B
A
MMPE
[methyl-3H] choline
(µM x 10-5)
DMPE
PC
Bs2WT
Bs2ΔchoX1ΔchoX2
Bs2ΔpmtAΔchoX1
ΔchoX2
1 2 3
Front
OL+PG+PE
UL
Start
Supplementary Figure S7. Analysis of Bs2WT, Bs2ΔchoX1ΔchoX2 and Bs2ΔpmtAΔchoX1ΔchoX2 after in vivo labelling with [methyl-3H] choline in mGSM. HPTLC revealed by charring (Panel A) 1: Bs2WT, 2: Bs2ΔchoX2ΔchoX1 and 3: Bs2ΔpmtAΔchoX1ΔchoX2 . Quantification of [methyl-3H] choline-labelled lipids in the PC positions in the HPTLC plate (Panel B). OL, ornithine lipids; PG, phosphatidylglycerol; PE, phosphatidylethanolamine; MMPE, monomethyl-phosphatidylethanolamine; DMPE, dimethyl-phosphatidylethanolamine; PC, phosphatidylcholine; UL, unknown lipid.
